# Antiviral Activity of Benzoheterocyclic Compounds from Soil-Derived *Streptomyces jiujiangensis* NBERC-24992

**DOI:** 10.3390/molecules28020878

**Published:** 2023-01-15

**Authors:** Manli Liu, Mengyao Ren, Yani Zhang, Zhongyi Wan, Yueyin Wang, Zhaoyuan Wu, Kaimei Wang, Wei Fang, Xiliang Yang

**Affiliations:** 1 Hubei Biopesticide Engineering Research Centre, Hubei Academy of Agricultural Sciences, No. 8, Nanhu Ave., Hongshan District, Wuhan 430064, China; 2 Institute of Infection, Immunology and Tumor Microenvironment, Institute of Pharmaceutical Process, Department of Pharmacy, Hubei Province Key Laboratory of Occupational Hazard Identification and Control, School of Medicine, Wuhan University of Science and Technology, No. 2, Huangjiahu East Road, Hongshan District, Wuhan 430081, China

**Keywords:** heterocyclic, virantmycin, PRV, *Streptomyces*, antiviral activity

## Abstract

Pseudorabies virus (PRV) is a pathogen that causes Aujeszky’s disease (AD) in animals, leading to huge economic losses to swine farms. In order to discover anti-PRV compounds, we studied the extracts of the strain *Streptomyces jiujiangensis* NBERC-24992, which showed significant anti-PRV activity. Eight benzoheterocyclic secondary metabolites, including three new compounds (**1**–**3**, virantmycins D–G) and five known compounds (**4**–**8**, virantmycin, A-503451 D, A-503451 D acetylate, A-503451 A, and A-503451 B), were isolated from the broth of NBERC-24992. The structures of the new compounds were identified by using extensive spectroscopic data, including mass spectrometry (MS), nuclear magnetic resonance (NMR), and electronic circular dichroism (ECD). Compound **1** was found to be a novel heterocyclic compound with a tricyclic skeleton from natural product. All compounds were tested for antiviral activity, and **4** (virantmycin) showed an excellent effect against PRV and was better than ribavirin and acyclovir. Our study revealed that chlorine atom and tetrahydroquinoline skeleton were important active moiety for antiviral activity. Virantmycin could be a suitable leading compound for an antiviral drug against PRV.

## 1. Introduction

Pseudorabies virus (PRV), also named suid herpes virus type 1, is a pathogen that causes Aujeszky’s disease (AD) in animals [1,2]. The symptoms of AD are similar to rabies, including fever, dyspnea, respiratory problems, neurological disorders, and abortions [3]. All aged pigs, as the natural host, are susceptible to be infected by PRV, which brings huge economic losses to swine farms [4,5]. Although PRV cannot infect humans, some cases have been reported in humans, indicating the risk of PRV transmission from animals to humans [6,7,8]. Vaccination is the most important methods to control PRV; with the widespread application of vaccines, PRV has even been eradicated from pig farms in some developed country [9,10,11]. However, a lot of outbreaks of AD have been found in Chinese swine farms, indicating PRV is still a potential threat for the pig industry in China [12]. With the emergence of novel antigenic variant PRV, the conventional vaccines do not prevent PRV infection in China [13]. A new vaccine or a more efficient drug is now necessary to control PRV.

At present, no anti-PRV drugs have been approved, and, thus, the discovery of highly active compounds is required. Actinomycetes are Gram-positive bacteria that belong to the unique group of prokaryotic microorganisms which form mycelia and spores, such as fungi [14]. Actinomycetes are a fruitful source of bioactive compounds, from which two-thirds of all naturally derived antibiotics and many anticancer, antihelminthic, antifungal, and immunosuppressive drugs are derived [15,16]. In our ongoing search for the antiviral compounds from actinomycetes, the ethyl acetate (EtOAc) extracts of the strain *Streptomyces jiujiangensis* NBERC-24992 showed significant anti-PRV activity, and eight benzoheterocyclic secondary metabolites were isolated from the EtOAc extracts. Herein, we report the isolation, structure elucidation, and anti-PRV activity of compounds from the actinomycetes NBERC-24992.

## 2. Results

**Figure 1:**
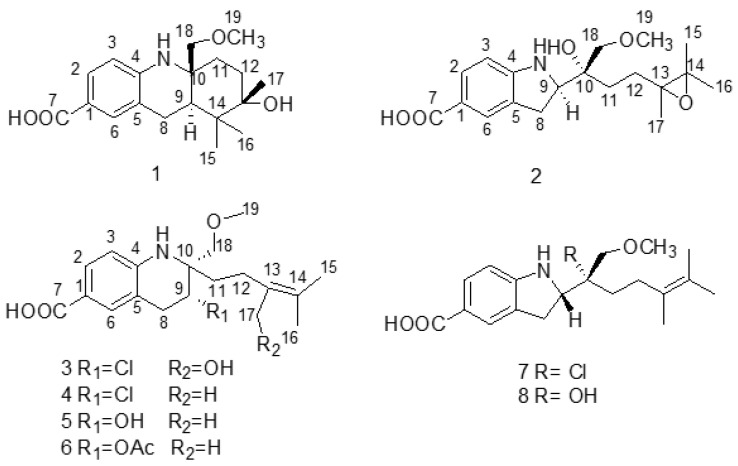
The structure of compounds **1**–**8**.

In the chemical investigation of the EtOAc extract, eight compounds, including three benzopyrrole derivatives and five benzopyridine derivatives, were isolated and identified. There are three new compounds (**1–3**, virantmycin D–G) and five known compounds (virantmycin **4** [17], A-503451 D **5 [18]**, A-503451 D acetylate **6** [18], A-503451 A **7 [18]**, and A-503451 B **8 [18]**) (Figure 1).

Compound **1** was obtained as a white amorphous powder. The molecular formula is presented as C_19_H_27_NO_4_ by HRESIMS (m/z 356.1838 [M + Na]^+^, Figure S7), corresponding to seven degrees of unsaturation. The ^1^H NMR spectrum shows three aromatic signals *δ* 7.62 (s), *δ* 7.57 (d, *J* = 8.4 Hz), and *δ* 6.50 (d, *J* = 8.4 Hz); one methoxyl signal *δ* 3.27 (s); and three methyl signals *δ* 1.19 (s), *δ* 1.03 (s), and *δ* 0.90 (s). The ^13^C NMR and DEPT spectra of **1** presents 19 carbon signals, including seven quaternary carbons (one carbonyl and three aromatic signals), four methine carbons (three aromatic signals), four methylene carbons, and four methyl carbons. One carbonyl (*δ* 169.9) and six aromatic (*δ* 148.4, 131.4, 128.8, 119.6, 117.2, and 112.8) signals are displayed in the low field of the ^13^C NMR spectrum, indicating the presence of one aromatic system and one carbonyl group for five degrees of unsaturation. Thus, **1** has two other ring structures, besides benzene ring.

**Figure 2:**
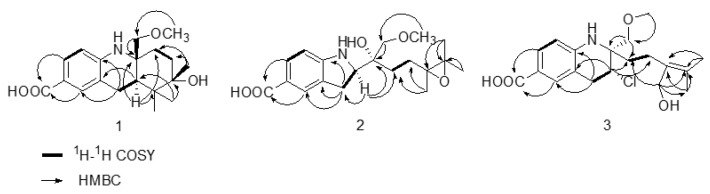
The key ^1^H-^1^H COSY and HMBC of compounds **1**–**3**.

In the ^1^H-^1^H COSY spectrum, the correlations from H-6 to H-2, from H-2 to H-3, from H-8 to H-9, and from H-11 to H-12 are observed (Figure 2). Compound **1** has a benzopyridine skeleton as deduced by the correlations between H-8 and C-4, between H-8 and C-5, between H-8 and C-6, between H-8 and C-10, and between H-9 and C-5 in the HMBC spectrum and the chemical shift value of C-10 (*δ* 54.5) and C-4 (*δ* 143.4) [17]. C-14 is connected to C-9 as determined by the correlations from H-9 to C-14, from H-9 to C-15, from H-9 to C-16, from H-8 to C-14, from H-15 to C-9, and from H-16 to C-9 in the HMBC spectrum. The correlations from H-2 to C-7 and from H-6 to C-7 show that the carbonyl group is located at C-6. The methoxyl group is linked to C-18 as deduced by the correlations between H-19 and C-18 and between H-18 and C-19. The substituent position of the hydroxyl is suggested to be C-13 based on the correlations between H-11 and C-13, between H-12 and C-13, between H-15 and C-13, between H-16 and C-13, and between H-17 to C-13. The structure of **1** is determined based on the ^1^H-^1^H COSY, HSQC, and HMBC spectra, as shown in Figure 2.

**Figure 3:**
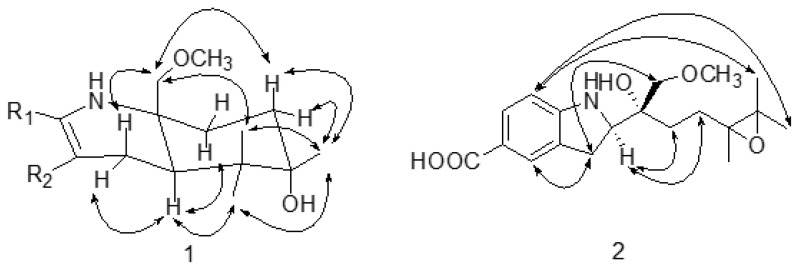
The key ROESY correlations of compounds **1**–**2**.

**Figure 4:**
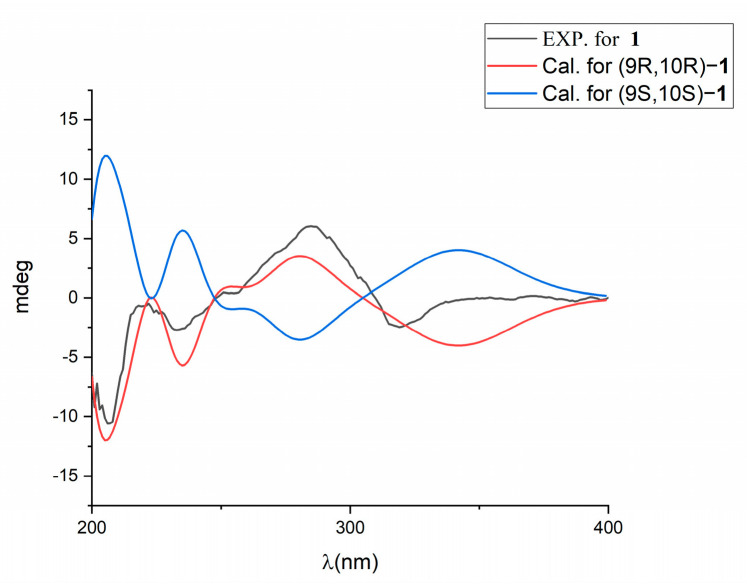
Comparison between the calculated and experimental ECD spectra of **1**.

The coupling constants of H-9 (dd, *J* = 14.4, 4.8 Hz) suggest that H-9 is axial. In the ROESY spectrum, the correlations between H-9 and H-8_eq_ (*δ* 2.64), between H-9 and H-15 (*δ* 1.03), and between H-9 and H-11_ax_ (*δ* 1.74) are observed (Figure 3). The correlations from H-18 to H-8_ax_ (*δ* 2.72), from H-18 to H-16 (*δ* 0.90), and from H-18 to H-12_ax_ (*δ* 1.85) indicate that the orientation of C-18 is axial (Figure 3). The correlations between H-17 and H-12_ax_ (*δ* 1.85), between H-17 and H-12_eq_ (*δ* 1.48), between H-17 and H-15 (*δ* 1.03), and between H-17 and H-16 (*δ* 0.90) suggest that the methyl of C-13 is equatorial. The CD spectrum shows that the positive signal at 285 nm and the negative signal at 318 nm are similar to those of (+)-virantmycin [19], and the calculated ECD curve for 9R and 10R is similar to the measured curve (Figure 4), indicating that the configurations of position C-9 and C-10 are R and R. Thus, the absolute configuration of **1** is assigned to be 9R, 10R, 13S, and it is named virantmycin D.

The molecular formula of compound **2** is determined to be C_19_H_27_NO_5_ by using HRESIMS (m/z 372.1787 [M + Na] ^+^, Figure S16), suggesting that **2** has seven degrees of unsaturation. The ^1^H NMR signals of **2** show three aromatic protons *δ* 7.83 (d, *J* = 8.4 Hz), 7.63 (s), and 7.61 (d, *J* = 8.4 Hz); one methine group *δ* 3.60 (t, *J* = 8.4 Hz); three methylene group; one methoxyl group *δ* 3.29 (s); and three methyl groups *δ* 1.39 (s), *δ* 1.28 (s), and *δ* 1.24 (s). Compound **2** is regarded as an analogue of virantmycin because of similar UV characteristics (Figure S17). The coupling constants of H-9 (t, *J* = 8.4 Hz) and the chemical shift value of C-9 (*δ* 63.4) and C-10 (*δ* 71.0) reveal that **2** has a benzopyrrole structure [20]. The molecular weight of **2** is 16 amu more than that of A-503451 B, and the main difference in the ^13^C-NMR data between **2** and A-503451 B is the loss of two olefinic signals and the addition of two oxygenated signals (*δ* 75.4 and *δ* 76.5), indicating the existence of epoxy group at position C-13 and C-14. In the HMBC spectrum, the correlations between H-15 and C-13, between H-15 and C-14, between H-11 and C-13, between H-12 and C-13, and between H-12 and C-14 confirm the epoxy moiety at C-13 and C-14. The planar structure of **2** was elucidated from the ^1^H-^1^H COSY, HSQC, and HMBC spectra (Figure 2).

**Figure 5:**
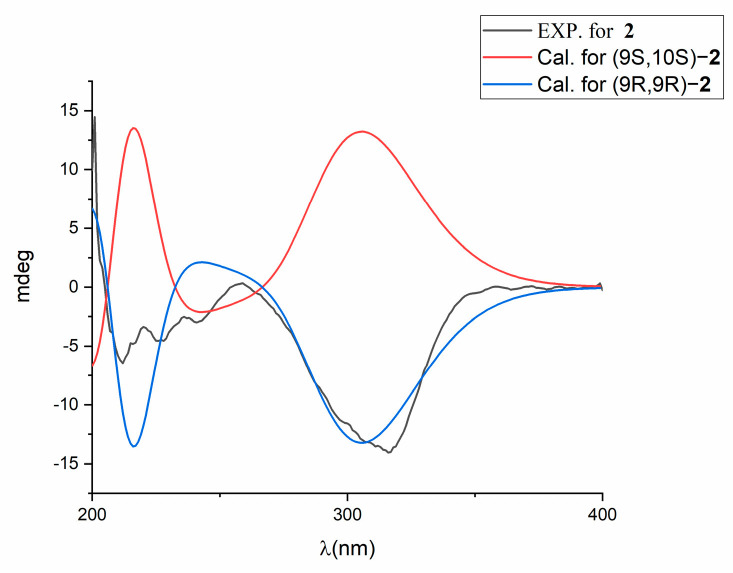
Comparison between the calculated and experimental ECD spectra of **2**.

In the ROESY spectrum, the correlations between H-3 and H-15, between H-3 and H-16, and between H-3 and H-17 support the spatial proximity between H-3 and the epoxy group (Figure 3), which is consistent with the upshift of H-3 (*δ* 7.83 for **2**, and *δ* 6.56 for A-503451 B). The correlations from H-9 to H-11b, from H-9 to H-12b, from H-8 to H-18, and from H-8 to H-9 in the ROESY spectrum suggest that the relative configuration of **2** is 9R^*^, 10R^*^ [20]. The absolute configuration at C-9 is speculated to be R, based on the different chemical shift values of H-8 and H-9 between **2** (H-9 (*δ* 3.60) and H-8 (*δ* 2.88)) and A-503451 B (H-9 (*δ* 4.17), H-8a (*δ* 3.17), and H-8b (*δ* 2.98)). In order to confirm the absolute configuration of C-9, a theoretical ECD calculation of 9R, 10R and 9S, 10S was applied. The calculated ECD curve for 9R, 10R is similar to the measured curve, indicating the configuration of C-9 is R (Figure 5). After failing to cultivate single crystal, the stereochemistry of C-13 was ambiguity. Thus, the absolute configuration of compound **2** is determined to be 9R, 10R, and it is named virantmycin E.

Compound **3** was isolated as a white amorphous powder. The molecular formula of compound **3** is deduced to be C_19_H_26_ClNO_4_ based on the HRESIMS (m/z 390.1441 [M + Na]^+^, Figure S24), which has one more oxygen atom than virantmycin [17]. The ^1^H and ^13^C NMR spectra (Table 1 and Table 2) are closely similar to those of virantmycin, suggesting that **3** is a derivative of virantmycin. The ^1^H NMR spectrum shows an additional methylene group *δ* 4.02 (s) and a missing methyl signal, which supports that one methyl group is oxidated in the structure. The HMBC correlations between H-17 and C-11, between H-17 and C-13, between H-17 and C-14, and between H-17 and C-15 confirm that the hydroxyl group is located at C-13. When combined with the 2D NMR spectra, including the ^1^H-^1^H COSY, HSQC, and HMBC spectra (Figure 2), the structure of **3** was identified, as shown in Figure 1. Compound **3** possesses the same absolute configurations as virantmycin revealed in the similarity of the CD data (Figure S25), having a negative signal at 288 nm and a positive signal at 315 nm [19]. Accordingly, the stereo-configuration **3** is elucidated to be 9R, 10R, and it is named virantmycin F.

All isolated compounds were tested for anti-PRV activity and cytotoxicity, and the results are shown in Table 3. Compounds **3** and **7** are active against PRV with the IC_50_ equals to 1.74 and 6.46 μg/mL, respectively. The CC_50_ of **7** is 1.68 μg/mL and the SI index of **7** is less than one, indicating the antiviral activity of **7** depends on its toxicity. Compound **3** also has slightly cytotoxic effect at a concentration of 20 μg/mL (only 80% viable cells compared to blank group). Compound **4** does not show any toxic effect on the tested cells at 20 μg/mL and presents significant anti-PRV activity that is superior to the positive controls ribavirin and acyclovir. Compound **4** could protect the Vero cells against PRV, with more than 50% of cells being alive even when the concentration is diluted to 0.01 μg/mL. These results are similar to those reported in the literature [21].

**Figure 6:**
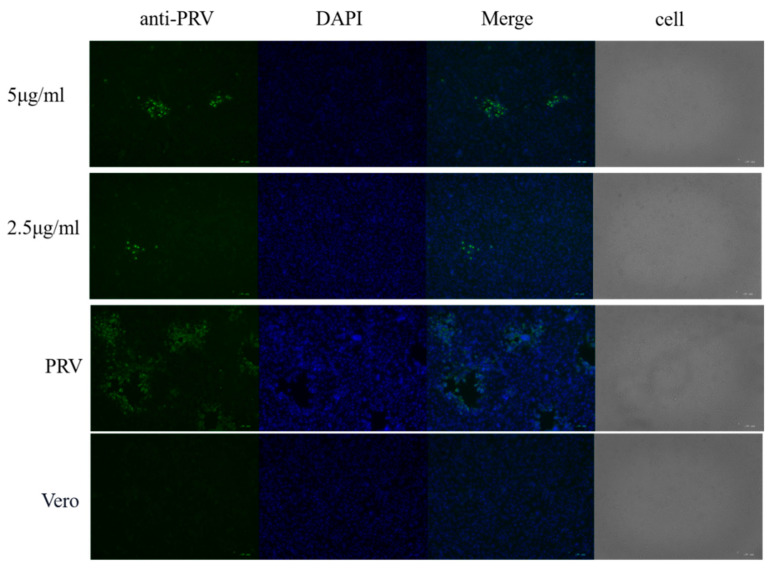
Indirect immunofluorescence assay of compound 2. Vero cells were infected with 100 TCID_50_ PRV for 1.5 h, and then compound 4 (5 and 2.5 μg/mL) was added. After 24 h, the indirect immunofluorescence assay was used for virus detection. The nucleocapsid (DAPI, blue) and anti-PRV (Green) responses are shown.

**Figure 7:**
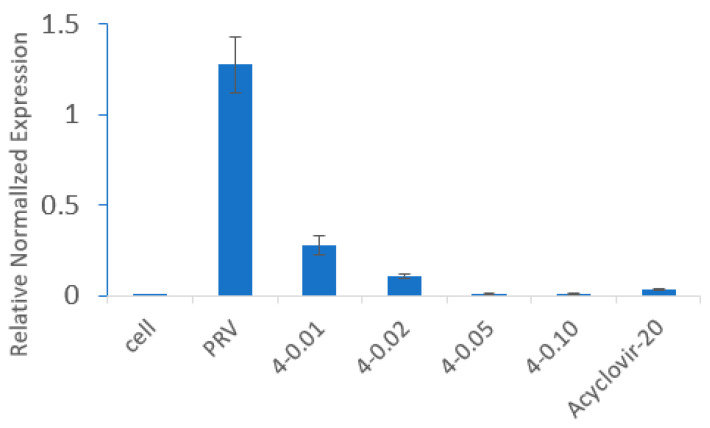
Compound 4 inhibits PRV proliferation. Vero cells were infected with 100 TCID_50_ PRV for 1.5 h, and then compound 4 (0.10, 0.05, 0.02, and 0.01 μg/mL) and acyclovir (20 μg/mL) were added. After 48 h incubation, cell suspension was collected, and DNA was extracted for qPCR.

In order to examine the post-treatment effect, Vero cells were infected with PRV for 1.5 h, and then the cells were treated with compound **4**. After 24 h infection, the results of an indirect immunofluorescence assay indicated that the virus infection in the PRV-infected cells was significantly reduced by compound **4** (Figure 6). After 48 h, the total DNA isolated from the supernatant was subjected to qPCR analysis by using PRV gE-specific primers. As shown in Figure 7, **4** inhibits PRV proliferation in a dose–dependent manner. These results demonstrate that **4** exhibits significant antiviral activity against PRV.

## 3. Discussion

**Figure 8:**
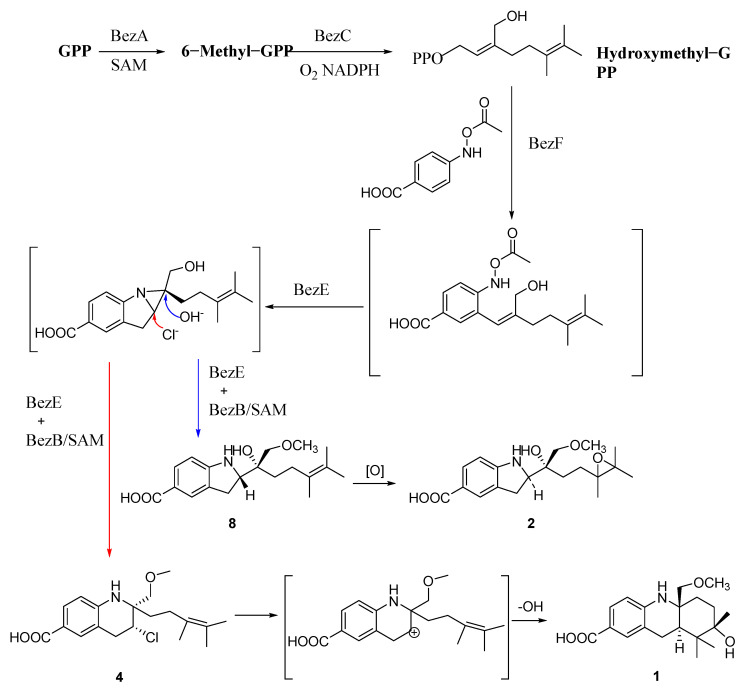
The probably biosynthetic process of compounds **1** and **2**.

Benzastatins or virantmycins are isolated from *Streptomyces* species of actinomycetes, which have unique heterocyclic structures that contain indoline or tetrahydroquinoline unit [17,18,21,22,23]. Those compounds were derived from geranyl diphosphate (GPP) and p-aminobenzoic acids. Then, the acetylated geranylated p-aminobenzoic acids (GPAA) were catalyzed to generate an aziridine ring by the key cytochrome P450 (BezE), and hydroxide ion or chloride ion attacked different position of the aziridine ring to form indoline or tetrahydroquinoline moiety through nucleophilic addition (Figure 7) [24]. Benzastatin E may be oxidized by monooxygenase to obtain compound **2** with an epoxy moiety at C-13 and C-14 [25]. Due to the structural stability of the indoline moiety, the configuration at C-9 is reversed. After the chlorine atom has left, **1** is possibly formatted by the cyclization of the electrophilic addition reaction, and the orientation of the methoxymethyl side chain changes during the annulation reaction (Figure 8).

Virantmycin, which was first isolated from the fermentation broth of *Streptomyces nitrosporeus*, has been reported to show significant antiviral activity against various RNA and DNA viruses [19,26]. The antiviral activities of virantmycin derivatives indicate that the configurations of the tetrahydroquinoline ring unit, the carboxylic group, and the terminal tetrasubstituted double bond are responsible for the anti-influenza virus activity [19]. Benzastatin C, an amide derivative of virantmycin, has specific activities against several viruses, and benzastatin D, a hydroxyl substitute of benzastatin C, is inactive against the tested viruses, which suggests that chloride plays an important role as the active moiety for antiviral activity [27]. The present study (Table 1) also indicates that the chlorine atom is necessary for the antiviral activity. The good leaving groups (for example, chlorine or mesyl group) in the structure are shown to be essential to the activity, and the compound may react with the target protein through the substitution reaction [18]. Compound **4** has the best activity among the isolated compounds, which leads to the conclusion that the tetrahydroquinoline skeleton and the methyl group of the terminal double bond play an important role in the activation of anti-PRV activity. Benzastatin C and benzastatin D show mostly equal free radical scavenging activity, indicating that their anti-PRV activity is not related to antioxidant ability [28]. The mode of action of virantmycin against PRV should be further investigated, and the effect of virantmycin on AD needs to be verified in vivo.

## 4. Materials and Methods

### 4.1. General Experimental Procedure

All solvents used in isolation were of analytical grade and obtained from Sinopharm group. Optical rotations: MPC 500 (Waltham) polarimeter (PerkinElmer Ltd., Waltham, MA, USA). Electronic circular dichroism (ECD) spectra: (Applied Photophysics Ltd., Leatherhead, UK). UV spectra: Shimadzu UV-2600 PC spectrometer (Shimadzu, J.P.). NMR spectra: Bruker 600 MHz spectrometer using TMS as standard (Bruker BioSpin Group, Rheinstetten, Germany). HR-ESI-MS spectra: Thermo Q-T of Micromass spectrometer (Thermo Electron Corporation, Waltham, MA, USA). Preparative HPLC: Waters 2525 pump, Waters 2998 DAD detector, and Waters 2767 Autopurifcation System (Waters Corporation, Milford, MA, USA).

### 4.2. Biological Material

The actinomycete NBERC-24992 was isolated from a rhizosphere soil sample collected from Liuyang County, Hunan Province, China, in November 2008. The NBERC-24992 was identified as *Streptomyces jiujiangensis* through an analysis of its morphological characteristics and 16S rRNA sequence. A voucher strain was preserved as NO. NBERC-24992 at Hubei Biopesticide Engineering Research Center, Hubei Academy of Agricultural Sciences, in Wuhan, China.

Vero cells (GDC0029) were purchased from the China Center for Type Culture Collection (CCTCC) and were grown in Dulbecco’s modified Eagle’s medium (DMEM) supplemented with 10% fetal bovine serum (FBS) (Gemini, CA, USAGermany), 100 μg/mL of streptomycin, and 100 IU/mL of penicillin at 37 °C in 5% CO_2_ atmosphere. PRV was obtained from Professor Tan Chen of Huazhong Agricultural University. The virus was propagated in Vero cells and stored at −80 °C until use.

### 4.3. Fermentation

The strain was grown on a seed medium (mannitol 10g, glucose 10g, malt extract 10g, yeast extract 5g, and calcium carbonate dissolved in 1 L water). The seed culture was prepared by inoculating the spores of the NBERC-24992 into 2 l of seed medium in 500 mL flasks and maintained in a shaking incubator (150 rpm) at 28 ^◦^C for five days. For the main culture, the seed culture (2 L) was then added into 25 L of liquid medium (glucose 10g, soluble starch 40g, cornmeal 15 g, soybean meal 25 g, peptone 2 g, and (NH_4_)_2_SO_4_ 0.5g dissolved in 1 L water) in a 50 L automatic fermentation tank. After cultivation for five days, the culture liquid was freeze-dried and extracted by EtOAc.

### 4.4. Extraction and Isolation

The ethyl acetate extract (55.0 g) was performed on a silica gel column using a gradient mixture of petroleum ether–ethyl acetate (15:1-0:1) as the eluent to produce 13 major fractions. Fr.7 was separated by Sephadex LH-20 using methanol to further purify, and 3 fractions (Fr.7.1-Fr.7.3) were obtained by combining the same parts according to HPLC behavior. Fr.7.2 was separated by a preparative HPLC using CH_3_CN (30–90%) as an eluent (24 mL/min) to afford compounds **4** (52.3 mg, t_R_ = 22.19 min), **6** (3.8 mg, t_R_ = 21.61 min), and **7** (3.5 mg, t_R_ = 23.14 min). Fr.8 was chromatographed on a silica gel column, eluted with petroleum ether–ethyl acetate (4:1-0:1), and yielded 3 fractions (Fr.8.1–8.3). Fr.8.2 and Fr.8.3 were combined and then further separated over preparative HPLC to obtain **1** (6.3 mg, t_R_ = 11.00 min) and **8** (5.7 mg, t_R_ = 13.42 min). Fr.9 was subjected to a silica gel column using petroleum ether–ethyl acetate (4:1-0:1) to provide four portions (Fr.9.1-Fr.9.4). Among them, Fr.9.2 was subjected to a Sephadex LH-20 (MeOH) to provide 4 portions. Fr.9.2.3 was eluted on a preparative HPLC column (7.5 mL/min) with 70% CH_3_CN to give compound **5** (19.3 mg, t_R_ = 13.93 min). Fr.10 was subjected to a preparative HPLC using 24% CH_3_CN to obtain compound **3** (8.6 mg, t_R_ = 10.06 min). Finally, Fr.11 was chromatographed on a silica gel column and then purified by a preparative HPLC (elute with CH_3_CN-H_2_O, gradient from 20:80 to 80:20; 24.0 mL/min) to give compound **2** (9.0 mg, t_R_ = 12.53 min).

Virantmycin D (**1**): white amorphous powder; [α]^25^_D_ −15.0° (*c* 0.001, MeOH); UV (MeOH) *λ*_max_ (log ε): 230 (4.02), 310 (4.41) nm; CD (*c* 0.90 mM, MeOH) *λ*_max_(Δε) 207nm (−3.55), 233 nm (−0.91), 285nm (+2.03), 318nm (−0.80); ^1^H NMR (600 MHz, CD_3_OD) and ^13^C NMR (150 MHz, CD_3_OD) data, see Table 1; HR-ESI-MS *m/z* 356.1838 [M + Na]^+^ (calcd for C_19_H_27_NO_4_Na, 356.1826).

Virantmycin E (**2**): white amorphous powder; [α]^25^_D_ −127.9° (*c* 0.001, MeOH); UV (MeOH) *λ*_max_ (log ε): 236 (3.98), 317 (4.44) nm; CD (*c* 0.86 mM, MeOH) *λ*_max_(Δε) 213 nm (−2.09), 316 nm (−4.94); ^1^H NMR (600 MHz, CD_3_OD) and ^13^C NMR (150 MHz, CD_3_OD) data, see Table 1; HR-ESI-MS *m/z* 372.1787 [M + Na]^+^ (calcd for C_19_H_27_NO_5_Na, 372.1787).

Virantmycin F (**3**): white amorphous powder; [α]^25^_D_ −11.2° (*c* 0.001, MeOH); UV (MeOH) *λ*_max_ (log ε): 230 (4.02), 318 (4.42) nm; CD (*c* 0.82 mM, MeOH) *λ*_max_(Δε) 210 nm (−1.60), 225 nm (−0.49), 249nm (+0.26), 288 nm (−1.10), 315 nm (+0.37); ^1^H NMR (600 MHz, CD_3_OD) and ^13^C NMR (150 MHz, CD_3_OD) data, see Table 1; HR-ESI-MS *m/z* 390.1441 [M + Na]^+^ (calcd for C_19_H_26_ClNO_4_Na, 390.1448).

### 4.5. Theoretical ECD Calculation

In general, conformational analyses were carried out via random searching in the Sybyl-X 2.0 (Tripos Associates Inc., St Louis, MO, USA) using the MMFF94S force field with an energy cutoff of 5 kcal/mol. Subsequently, geometry optimizations and frequency analyses were implemented at the B3LYP-D3(BJ)/6-31G* level in CPCM methanol using ORCA5.0.1 [29]. All conformers used for property calculations in this work were characterized to be stable point on potential energy surface (PES) with no imaginary frequencies. The excitation energies, the oscillator strengths, and the rotational strengths (velocity) of the first 60 excited states were calculated using the TD-DFT methodology at the PBE0/def2-TZVP level in CPCM methanol using ORCA5.0.1. The ECD spectra were simulated by overlapping Gaussian function (half the bandwidth at 1/e peak height, sigma = 0.30 for all) [30]. Gibbs free energies for conformers were determined by using thermal correction at the B3LYP-D3(BJ)/6-31G* level and electronic energies were evaluated at the wB97M-V/def2-TZVP level in CPCM methanol using ORCA5.0.1. To obtain the final spectra, the simulated spectra of the conformers were averaged according to the Boltzmann distribution theory and relative Gibbs free energy (∆G).

### 4.6. Antiviral and Cytotoxic Activities of Compounds ***1***–***8***

The compounds were dissolved in DMSO to 20 mg/mL for storage. The Vero cells with 80% confluence in 96-well plates were added with different concentrations of compounds (0.31, 0.625, 1.25, 2.5, 5, 10, and 20 μg/mL) in a DMEM medium (2% FBS) with or without PRV virus at 100 TCID_50_ (50% cell culture infectious dose). After 48 h post infection, the supernatant was discarded and the cells were washed twice. A MTT assay was used for cell viability calculation. The effective antiviral concentration (with PRV virus) and cytotoxicity concentration (without PRV virus) were expressed as EC_50_ and CC_50_, respectively. EC_50_ and CC_50_ were calculated by using SPSS 16.0 software, and each dilution had four replicates. The selectivity index (SI) was used as the result of EC_50_/CC_50_. Ribavirin and acyclovir were used as the positive controls.

### 4.7. Indirect Immunofluorescence Assay

The monolayers of Vero cells cultured in 24-well plates were treated with PRV for 1.5 h, the supernatant was discarded, and then compound **4** was added in 5 μg/mL and 2.5 μg/mL. After 24 h incubation, the cells were washed with PBS twice, fixed with cold absolute ethanol at 4 °C for 15 min, permeabilized with 0.1% Triton X-100 in PBS for 10 min, and blocked with 1% BSA in PBS for 1 h. The cells were then incubated with 1:200 anti-pseudorabies virus antibody in PBS containing 1% BSA at 4 °C overnight. After washing three times with PBS, the cells were stained with 1:2000 goat anti-rabbit IgG H&L. To show the nucleus of the cells, DAPI was used to stain the cellular DNA. The fluorescence images were observed using an inverted fluorescence microscope (Olympus IX73, Japan). Cell morphology (CPE) was also recorded to show virus proliferation in the cells by using an inverted microscope.

### 4.8. Compounds Treated with Vero Cells after Being Infected with PRV

The Vero cells were infected with PRV (100TCID_50_) for 1.5 h, the supernatant was discarded, and the cells were washed twice. Then, compound **4** in different concentrations was added. Cell suspension was collected after 48 h for DNA extraction and qPCR. Total DNA was extracted by using a TIANamp Genomic DNA kit according to the protocol. Amplification reactions were conducted using the PrimeScript RT Master Mix with a CFX96 Real-time PCR Detection System (BioRad). The gene-specific real-time primers were GTGATGACCCACAACGGGTCAGCTCCTTGATGACC (PRV gE) and GACAACAGCCTCAAGATCG (GAPDH). Then, qPCR was conducted at 95 °C for 3 min, for a total of 40 cycles at 95 °C for 5 s and at 60 °C for 30 s, and a final dissociation stage was conducted at 95 °C for 10 s and at 65 °C for 1 min. Each experiment was repeated at least twice, and the relative gene expression level was assessed using the ΔΔCt method. PRV gE gene was detected, and GAPDH served as an internal control to normalize the loading of the template.

## 5. Conclusions

Eight benzoheterocyclic secondary metabolites, including three new compounds (**1**–**3**, virantmycin D–G) and five known compounds (**4**–**8**), were isolated from the broth of *S. jiujiangensis* NBERC-24992. Compound **1** was found to be a novel heterocyclic compound with a tricyclic skeleton from natural product. Compound **4** (virantmycin) showed an excellent effect against PRV and was better than the positive controls ribavirin and acyclovir. Our study revealed that chlorine atom and tetrahydroquinoline skeleton were important active moieties for antiviral activity. Virantmycin could be a suitable leading compound for an antiviral drug against PRV.

## Figures and Tables

**Table 1:** The ^1^H-NMR (600 MHz, *J* in Hz) and ^13^C-NMR (150 MHz) spectroscopic data of compounds **1**–**3** (CD_3_OD).

Position	1		2		3	
	^1^H-NMR	^13^C-NMR	^1^H-NMR	^13^C-NMR	^1^H-NMR	^13^C-NMR
1		119.6		117.8		117.4
2	7.57, (d, 8.4)	128.8	7.61, (d, 8.4)	127.8	7.64, (d, 8.4)	129.3
3	6.50, (d, 8.4)	112.8	7.83, (d, 8.4)	117.6	6.62, (d, 8.4)	112.8
4		148.4		147.0		147.4
5		117.2		121.5		115.4
6	7.62, s	131.4	7.63, s	131.2	7.63, s	131.6
7		169.9		169.5		169.3
8	2.72, (dd, 16.2, 14.4) 2.64, (dd, 16.2, 4.8)	23.9	2.88, m	33.3	3.37, (dd, 16.8, 4.8) 3.06, (dd, 16.8, 4.8)	33.1
9	2.23, (dd, 14.4, 4.8)	42.7	3.60, (t, 8.4)	63.4	4.45, (t, 4.8)	56.9
10		54.5		71.0		57.4
11	1.97, m 1.74, m	28.2	2.29, m 2.04, m	29.3	1.69, m 1.60, m	32.7
12	1.85, m 1.48, m	31.9	2.30, m 1.72, m	35.9	2.22, m 2.09, m	27.8
13		73.6		75.4		131.7
14		39.3		76.5		127.9
15	1.03, s	22.6	1.39, s	28.9	1.69, s	62.3
16	0.90, s	19.4	1.24, s	24.6	1.69, s	14.7
17	1.19, s	23.8	1.28, s	20.7	4.02, s	16.6
18	3.60, (d, 9.6) 3.28, (d, 9.6)	71.1	3.52, (d, 9.6) 3.48, (d, 9.6)	72.7	3.61, (d, 9.6) 3.51, (d, 9.6)	73.2
19	3.27, s	58.4	3.29, s	58.0	3.40, s	58.2

**Table 2:** The HMBC and ROESY correlations of compounds **1–3** (CD_3_OD).

Position	1		2		3	
	HMBC	ROESY	HMBC	ROESY	HMBC	ROESY
1						
2	4,6,7	3	4,6,7	3	4,6,7	3
3	1,5	2	1,5	2,15,16,17	1,5	2
4						
5						
6	2,4,7,8	8	2,4,7,8	8	2,4,7,8	8
7						
8	4,5,6,9,10	9,16,18 9,15	4,5,6,9,10	9,18	4,5,6,9,10	9, 11, 18
9	8,10,14,15,16,18	8,11_ax_,15	8,10, 18	8,11,12	5, 8,10, 18	8,10,11,12,18
10						
11	9,10,12,13,18	18, 12_ax_, 12_eq_ 9, 12_eq_, 18	10,12,13,18	12, 18	10,12,13,18	12
12	10,11,13,14,17	16,17,18 11_ax_,11_eq_, 17	11,13,14,17	11,15,17	11,13,14,17	11,15,16,17
13						
14						
15	9,13,14,16	8,9,16,17	13,14,16	12, 16, 17	13,14,16	12, 16, 17
16	9,13,14,15	12_ax_,15, 17,18	13,14,15	12, 15, 17	13,14,15	12, 15, 17
17	12,13,14	12_ax_, 12_eq_, 15, 16	12,13,14	12, 15, 16	11,12,13,14	12, 15, 16
18	10,11,19	8_ax_, 11, 12 _ax_, 16	9, 10,11,19	8, 11	9, 10,11,19	8, 9, 11
19	18		18		18	

**Table 3:** Antiviral activity and cytotoxicity of compounds **1**–**8**.

	Antiviral Activity ^a^ EC_50_/μg/mL	Cytotoxicity ^b^ IC_50_/μg/mL	SI		Antiviral Activity ^a^ EC_50_/μg/mL	Cytotoxicity ^b^ IC_50_/μg/mL	SI
**1**	-	>20	-	**6**	-	>20	-
**2**	-	>20	-	**7**	6.46 ± 1.38	1.68 ± 0.13	0.26
**3**	1.74 ± 0.25	>20	>11.5	**8**	-	>20	-
**4**	<0.31	>20	>64.5	Ribavirin	4.44 ± 1.53	>20	>4.50
**5**	-	>20		Acyclovir	2.96 ± 1.48	>20	>6.75

^a^ EC_50_ is the concentration that the compounds inhibit virus-induced cytopathic effect by 50%. ^b^ CC_50_ is the concentration that the compounds kill 50% of the normal cells.

## Data Availability

Not applicable.

## Supplementary Materials

The following supporting information can be downloaded at https://www.mdpi.com/article/10.3390/molecules28020878/s1, the NMR, HRMS, CD, and UV spectra of compounds **1–3** (Figures S1–S26), and the NMR spectra of compounds **4**–**8** (Figures S27–S36).
