# Supplementary material for: Antiviral Activity of Benzoheterocyclic Compounds from Soil-Derived Streptomyces jiujiangensis NBERC-24992
Source: Molecules. 2023 Jan 15;28(2):878. doi: 10.3390/molecules28020878 (PMC9866866; doi:10.3390/molecules28020878)
Supplement: Supplementary file 1 [file molecules-28-00878-s001.zip › molecules-2083858-supplementary.pdf]

Supplementary information for

Antiviral activity of benzoheterocyclic compounds from soil-derived *Streptomyces jiujiangensis* NBERC-24992

Manli Liu[1†], Mengyao Ren[2†], Yanni Zhang[1], Zhongyi Wan[1], Yueyin Wang[1], Zhaoyuan Wu[1], Kaimei Wang[1], Wei Fang[1]*, Xiliang Yang[2]*

[1] Hubei Biopesticide Engineering Research Centre, Hubei Academy of Agricultural Sciences, No. 8, Nanhu Ave., Hongshan District Wuhan 430064, China

[2] Department of Pharmacy, Medical College, Wuhan University of Science and Technology, No. 2, Huangjiahu East Road, Hongshan District, Wuhan 430081, China

[†] These authors contributed equally to the manuscripts

* Correspondence: wei.fang@nberc.com (W.F.); yxlyxl117@163.com (X.Y.)

Figure legends

Figure S1 ^{1}H-NMR spectrum of 1

Figure S2 ^{13}C-NMR spectrum of 1

Figure S3 ^{1}H-^{1}H COSY spectrum of 1

Figure S4 ^{1}H-^{13}C HSQC spectrum of 1

Figure S5 ^{1}H-^{13}C HMBC spectrum of 1

Figure S6 ROESY spectrum of 1

Figure S7 HR-MS spectrum of 1

Figure S8 CD spectrum of 1

Figure S9 UV spectrum of 1

Figure S10 ^{1}H-NMR spectrum of 2

Figure S11 ^{13}C-NMR spectrum of 2

Figure S12 ^{1}H-^{1}H COSY spectrum of 2

Figure S13 ^{1}H-^{13}C HSQC spectrum of 2

Figure S14 ^{1}H-^{13}C HMBC spectrum of 2

Figure S15 ROESY spectrum of 2

Figure S16 HR-MS spectrum of 2

Figure S17 UV spectrum of 2

Figure S18 ^{1}H-NMR spectrum of 3

Figure S19 ^{13}C-NMR spectrum of 3

Figure S20 ^{1}H-^{1}H COSY spectrum of 3

Figure S21 ^{1}H-^{13}C HSQC spectrum of 3

Figure S22 ^{1}H-^{13}C HMBC spectrum of 3

Figure S23 ROESY spectrum of 3

Figure S24 HR-MS spectrum of 3

Figure S25 CD spectrum of 3

Figure S26 UV spectrum of 3

Figure S27 ^{1}H-NMR spectrum of 4

Figure S28 ^{13}C-NMR spectrum of 4

Figure S29 ^{1}H-NMR spectrum of 5

Figure S30 ^{13}C-NMR spectrum of 5

Figure S31 ^{1}H-NMR spectrum of 6

Figure S32 ^{13}C-NMR spectrum of 6

Figure S33 ^{1}H-NMR spectrum of 7

Figure S34 ^{13}C-NMR spectrum of 7

Figure S35 ^{1}H-NMR spectrum of 8

Figure S36 ^{13}C-NMR spectrum of 8

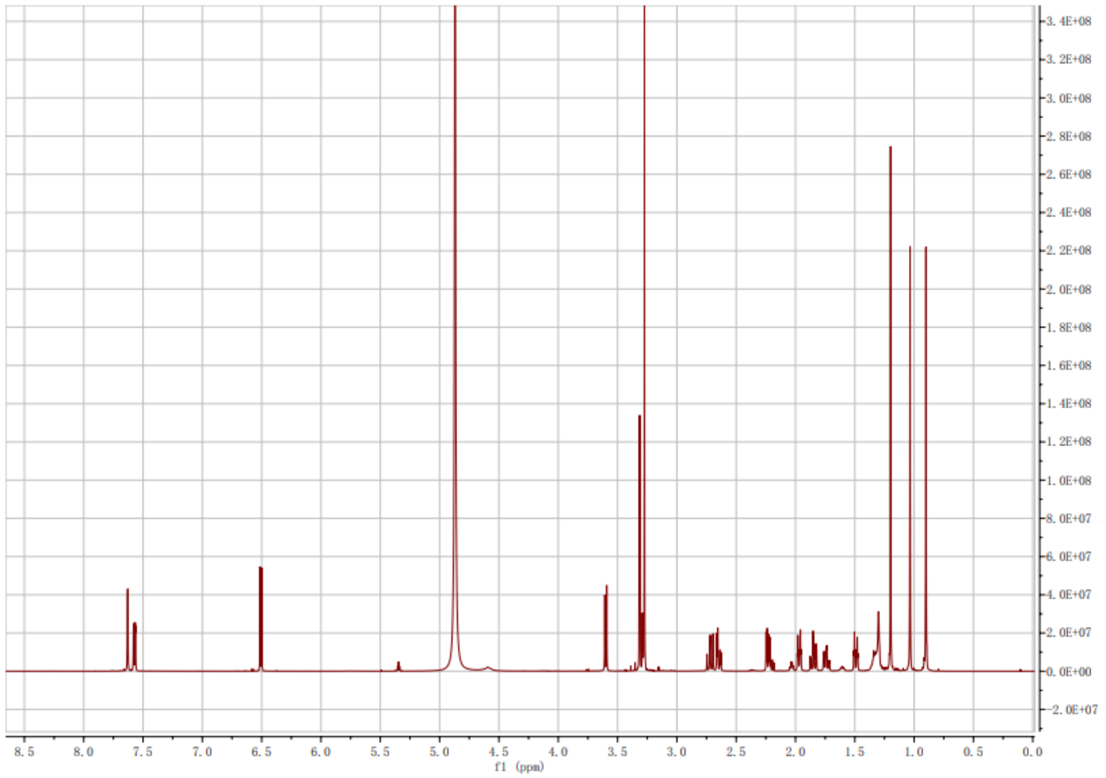

Figure S1

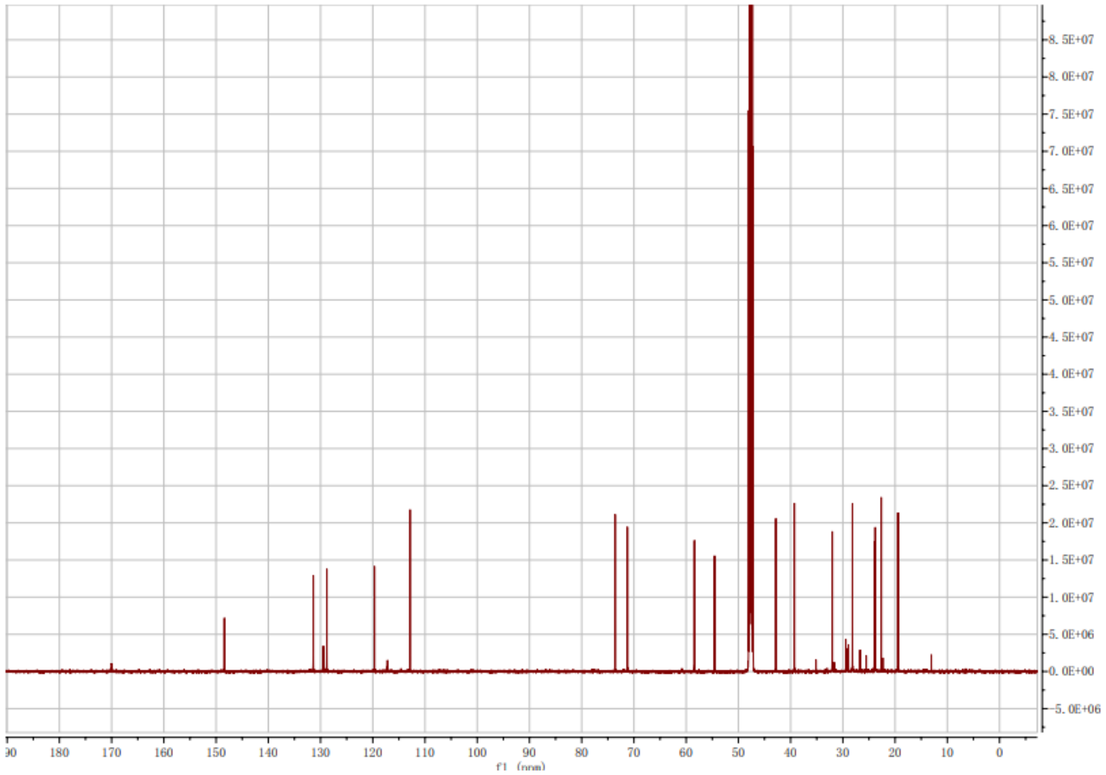

Figure S2

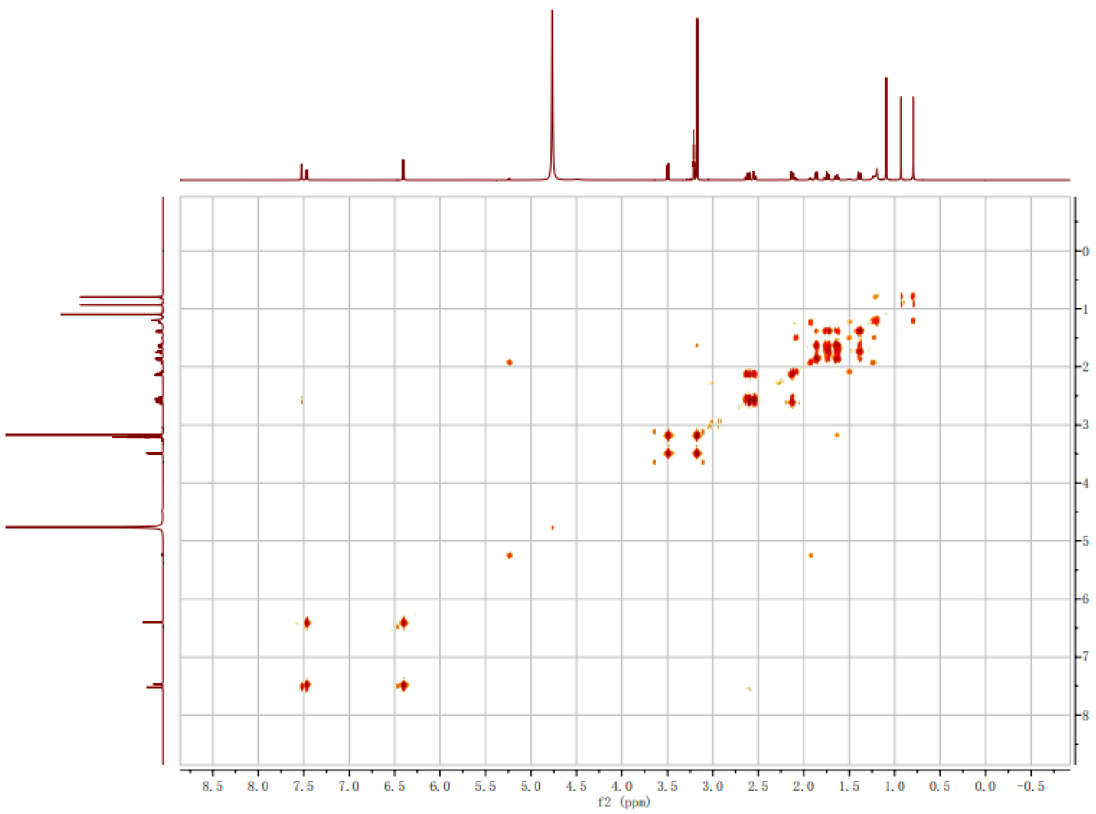

Figure S3

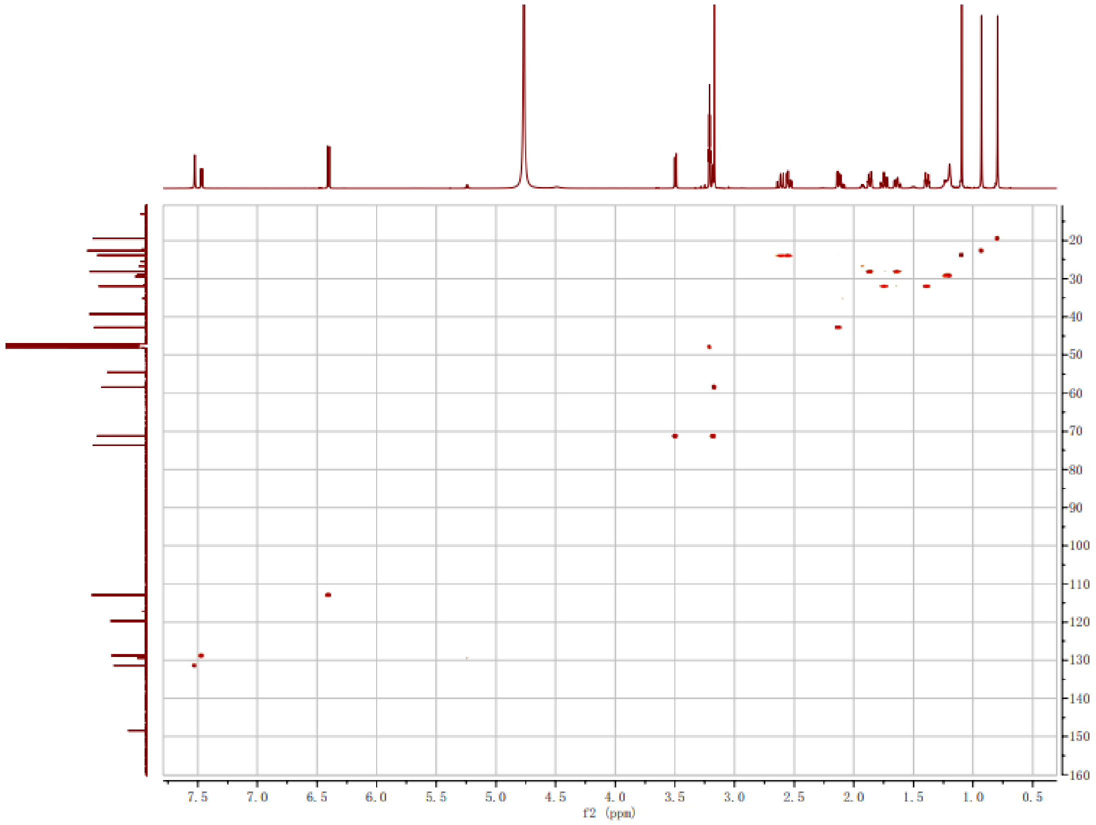

Figure S4

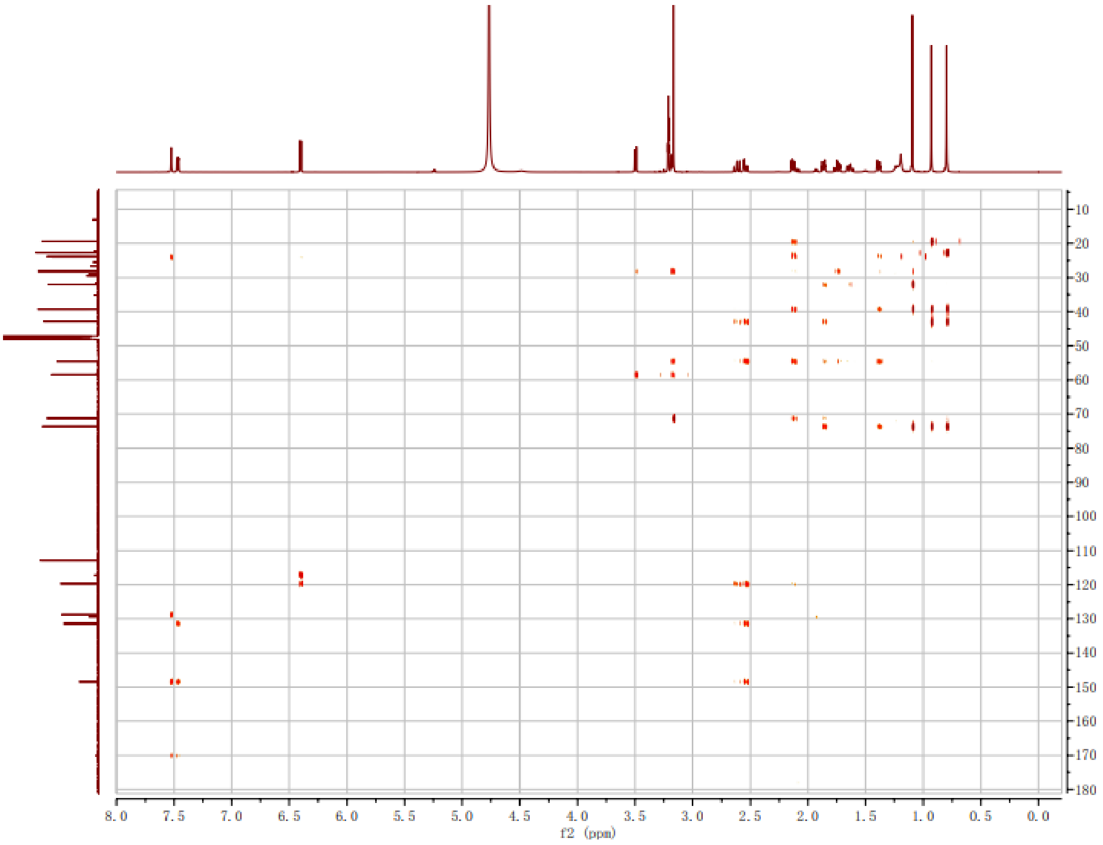

Figure S5

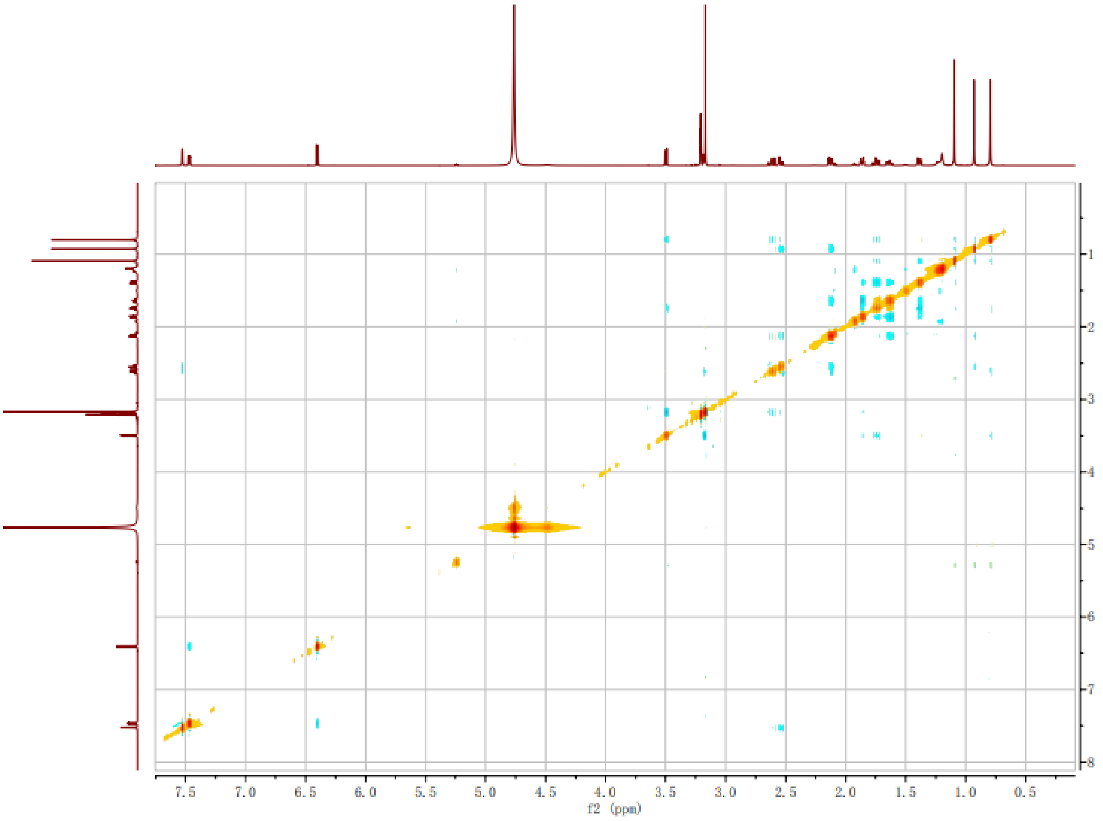

Figure S6

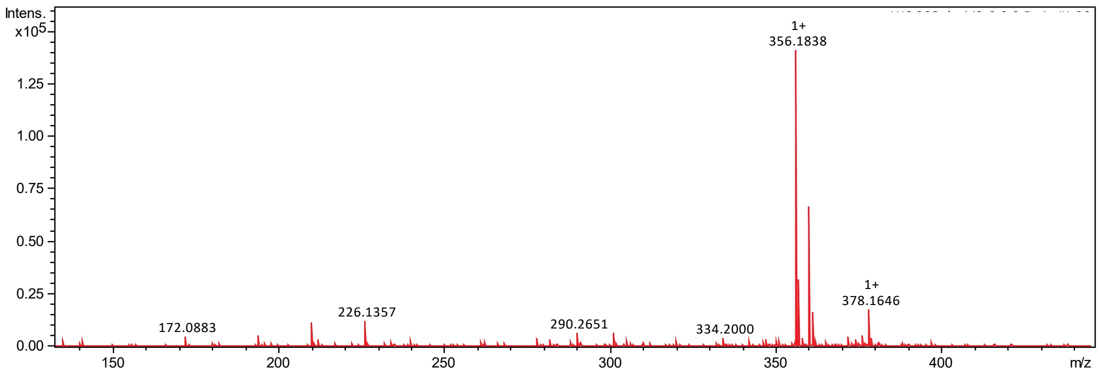

Figure S7

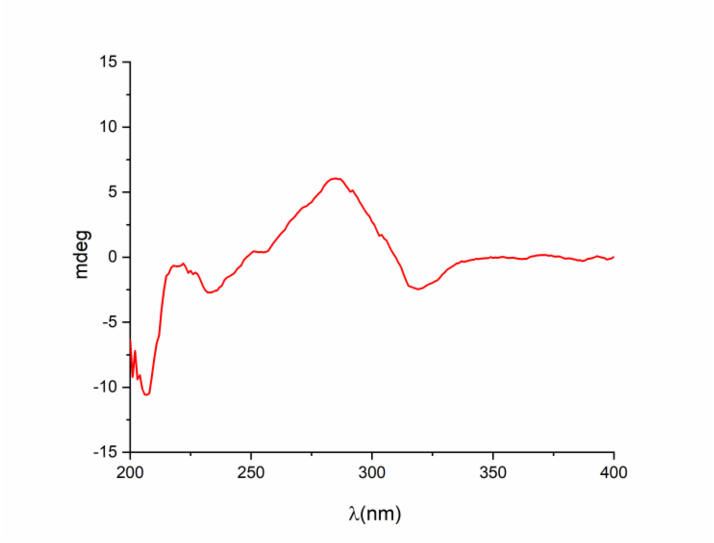

Figure S8

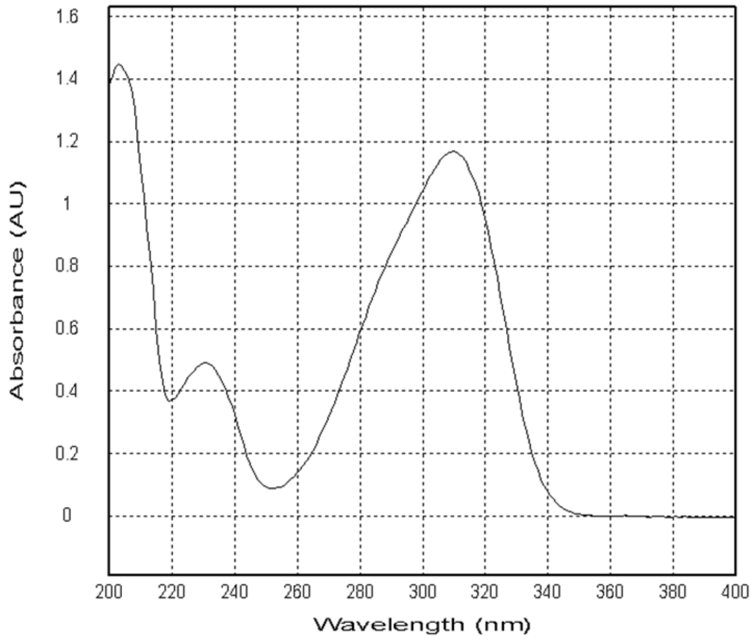

Figure S9

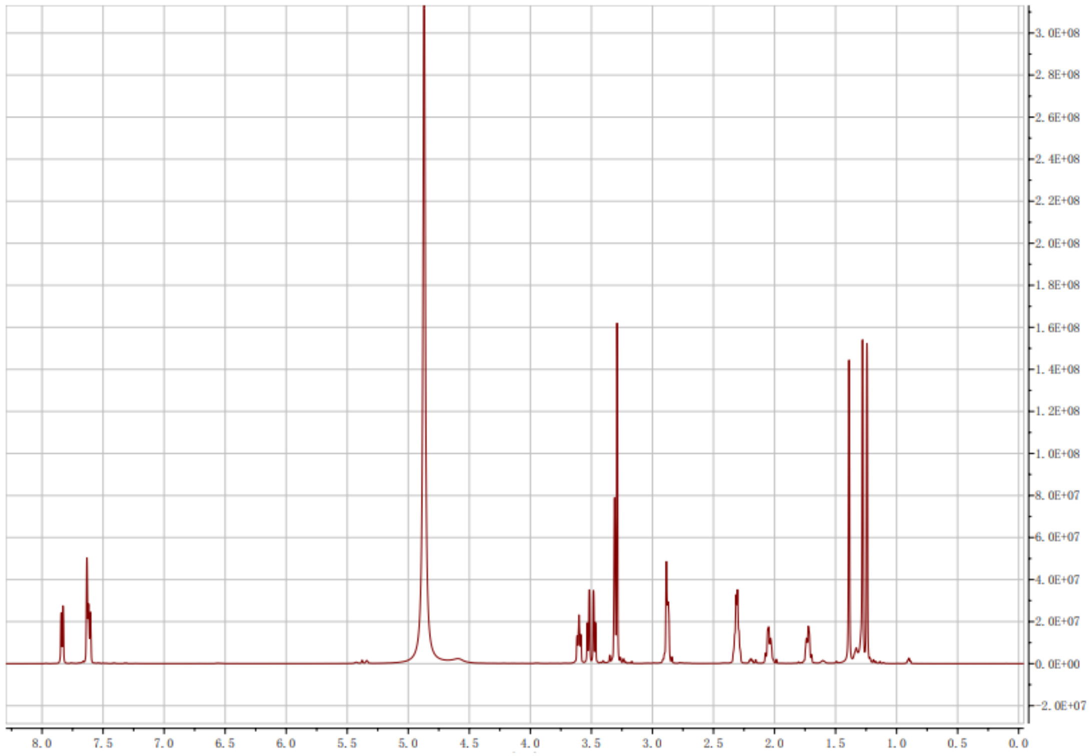

Figure S10

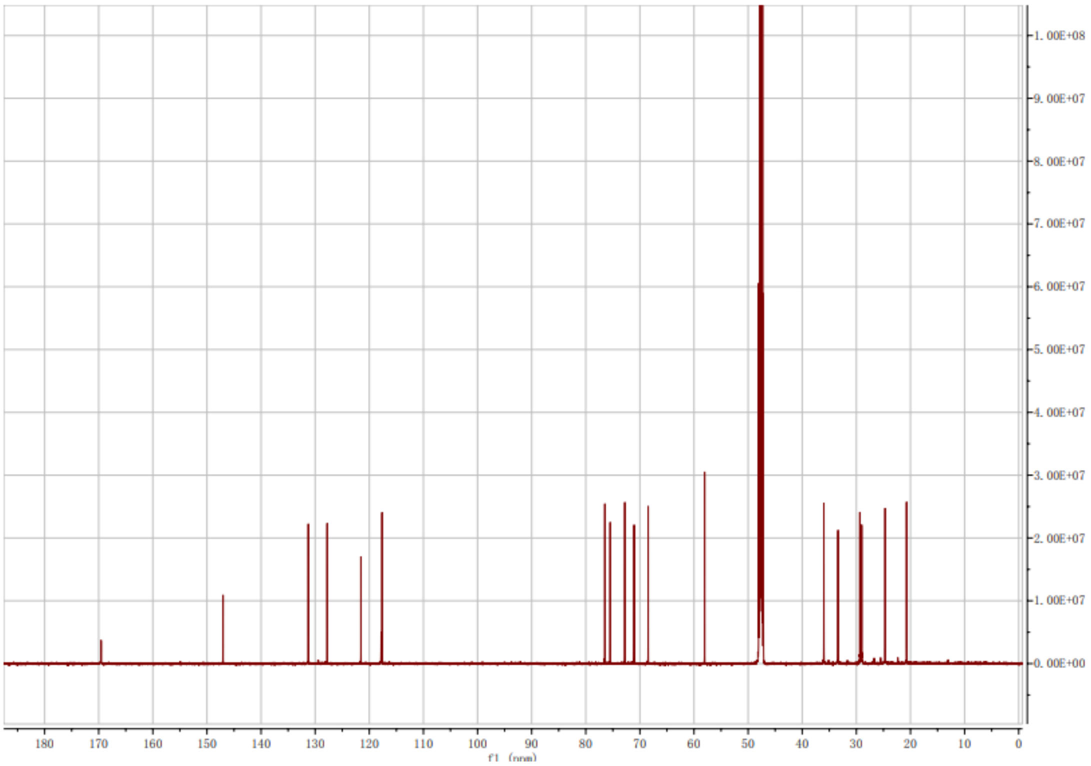

Figure S11

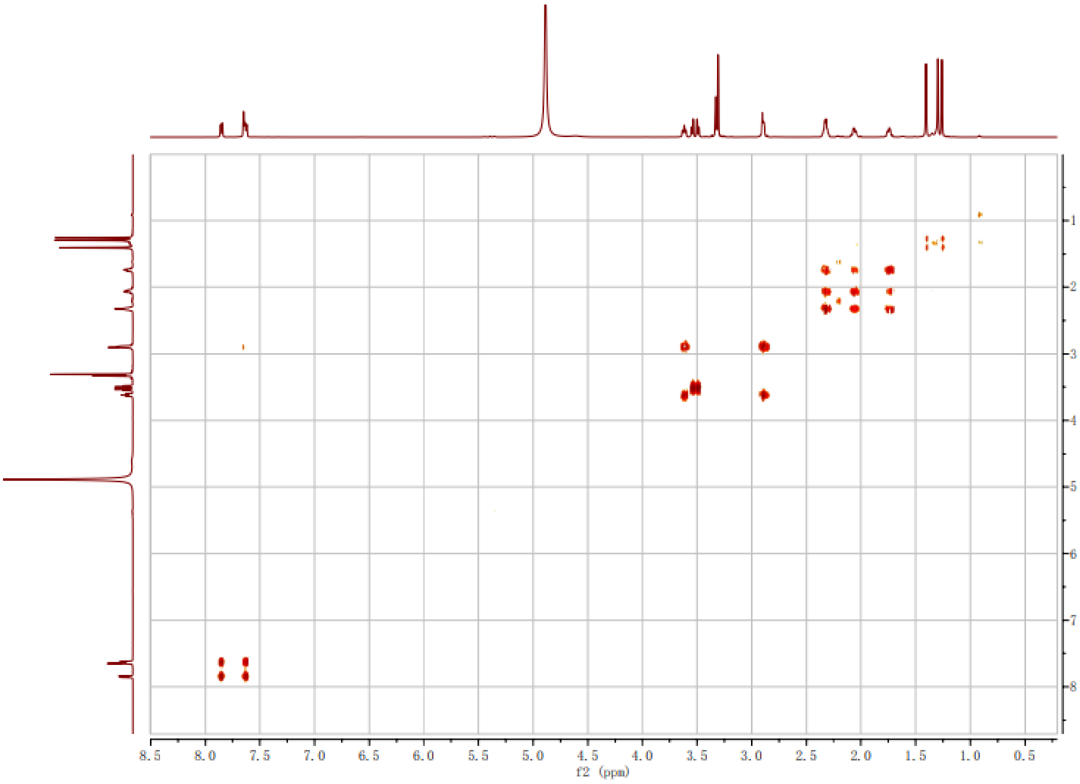

Figure S12

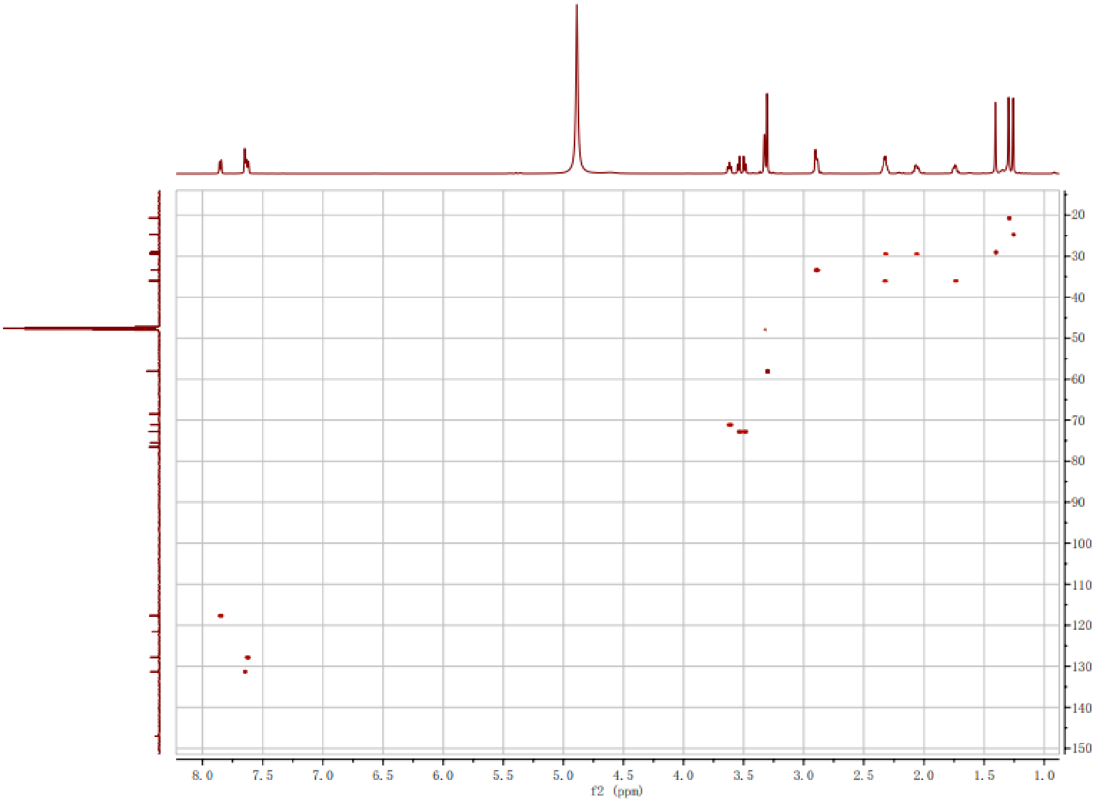

Figure S13

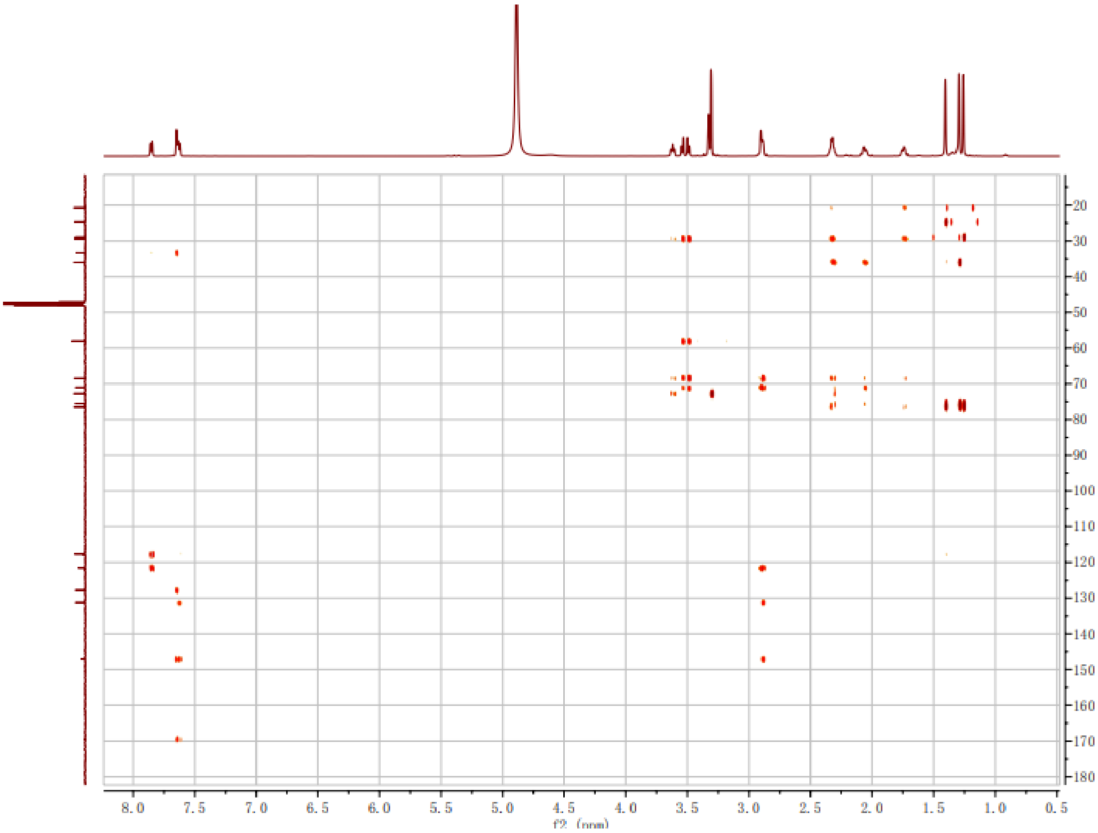

Figure S14

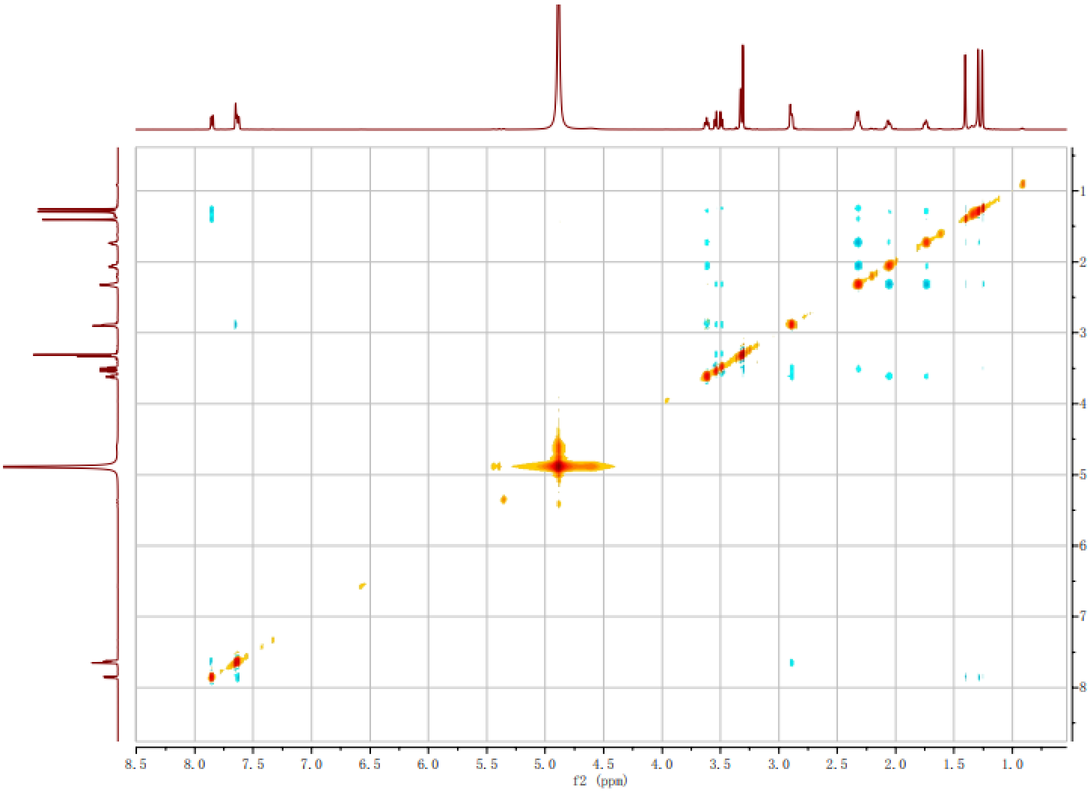

Figure S15

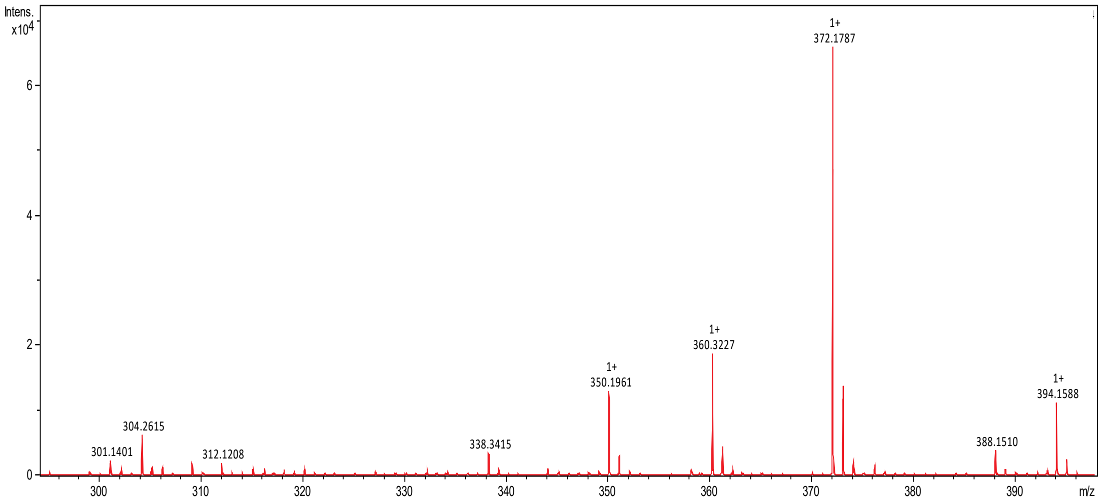

Figure S16

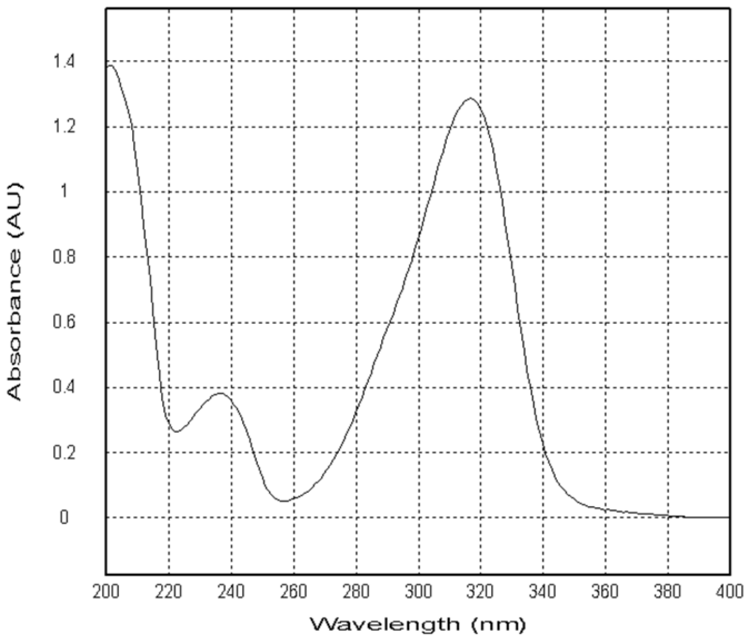

Figure S17

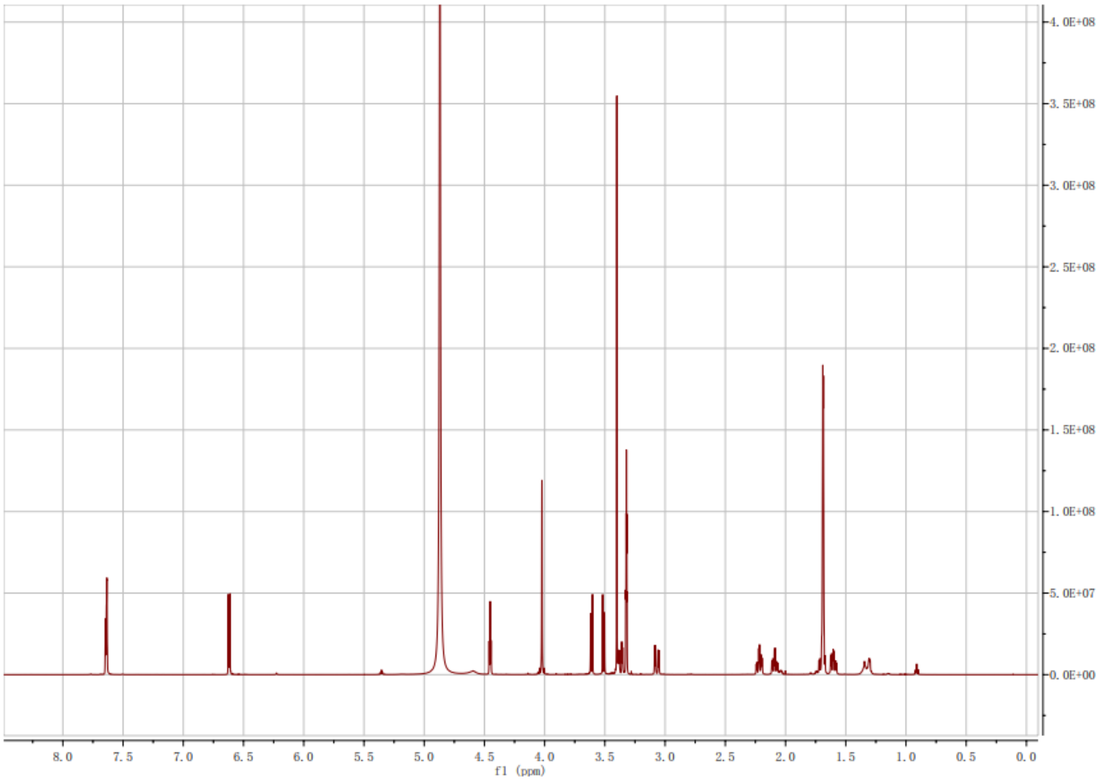

Figure S18

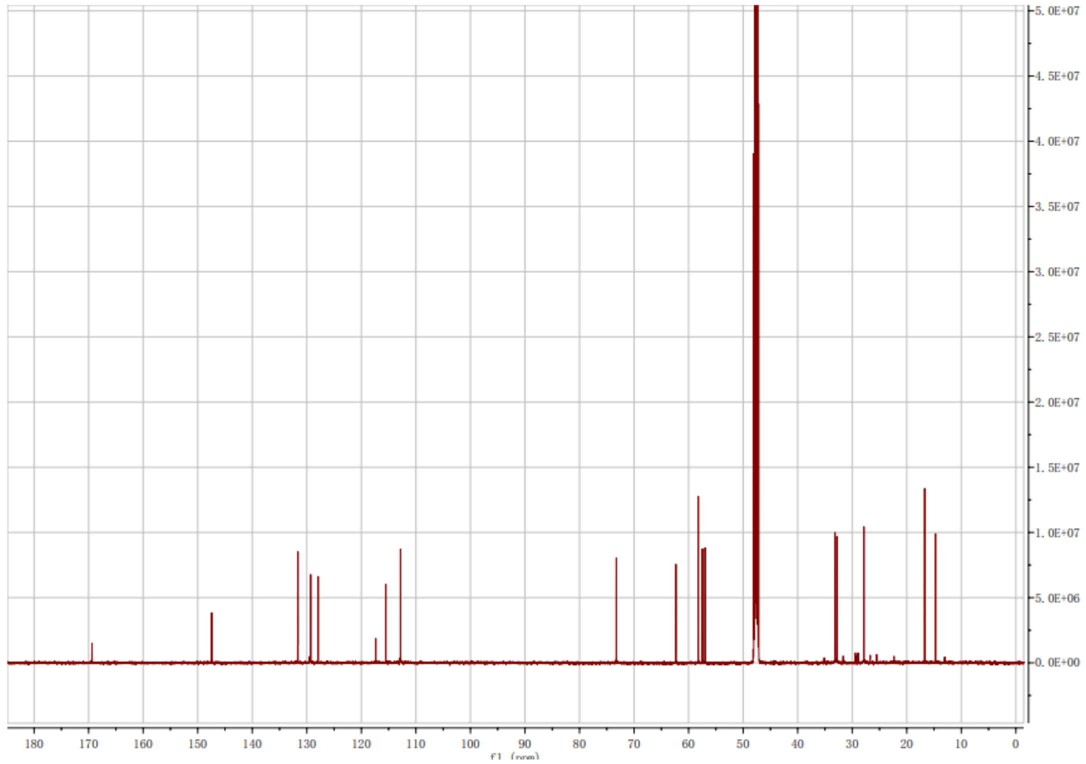

Figure S19

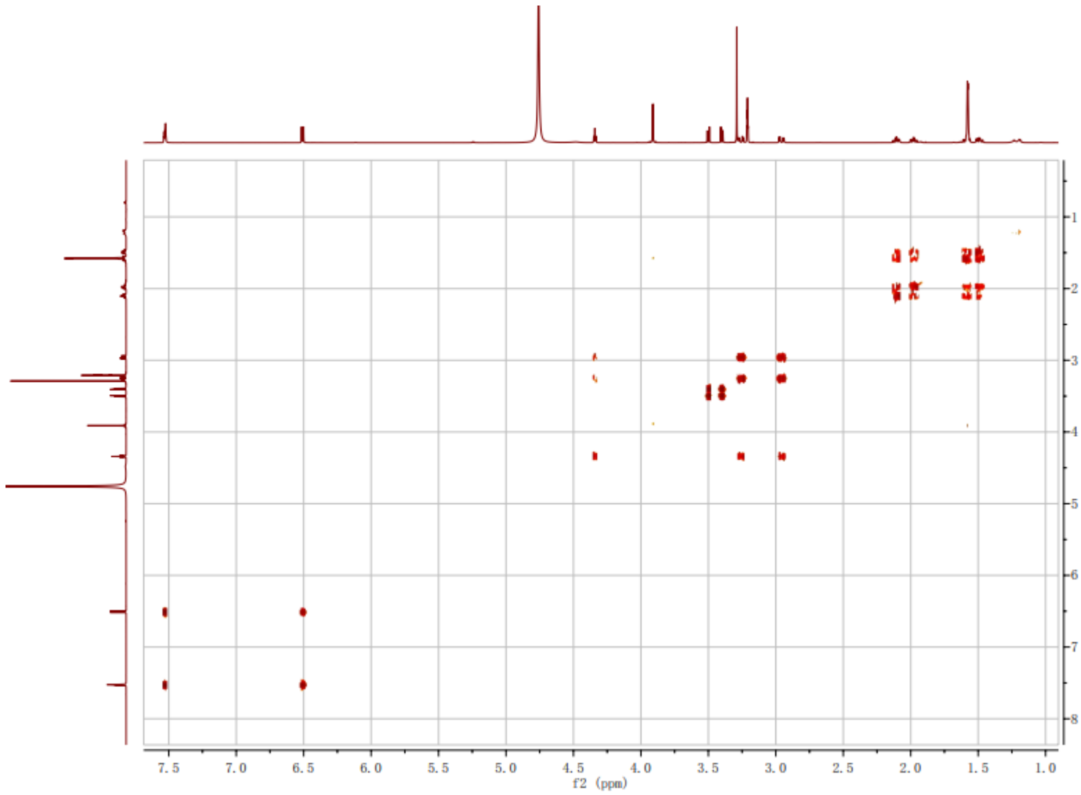

Figure S20

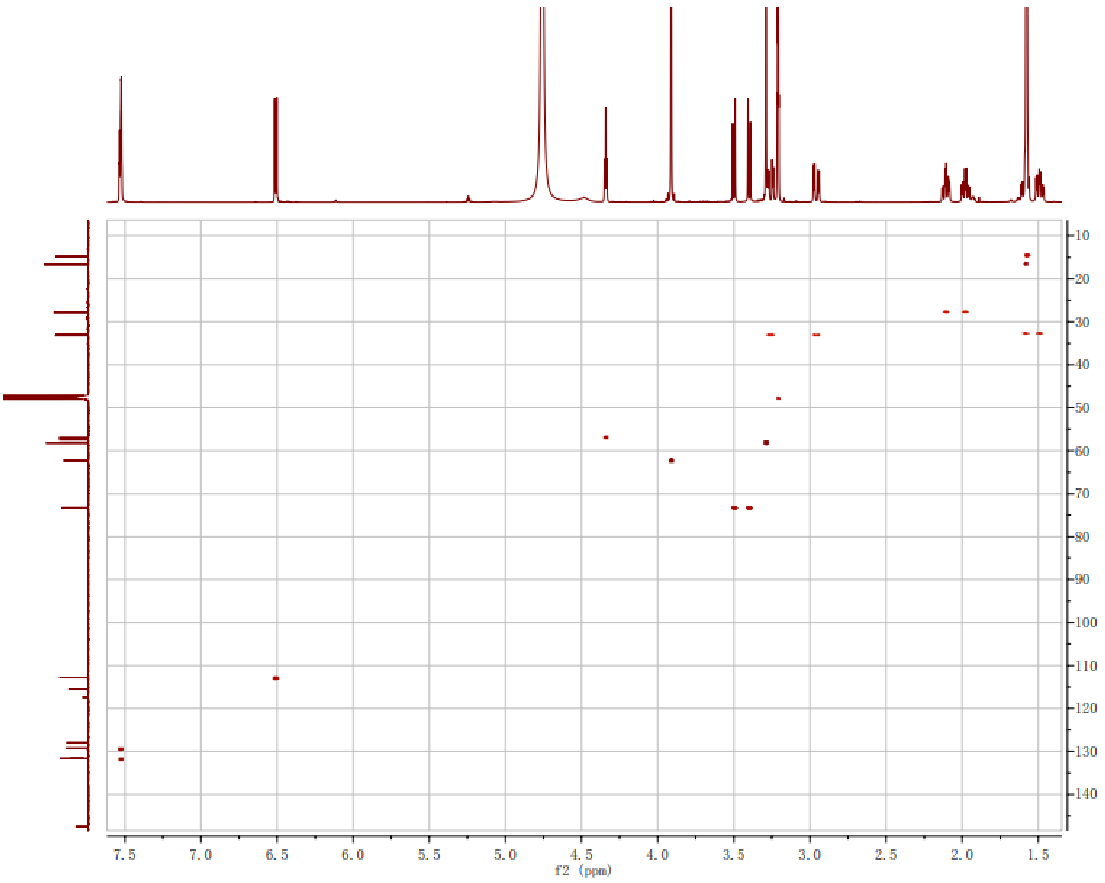

Figure S21

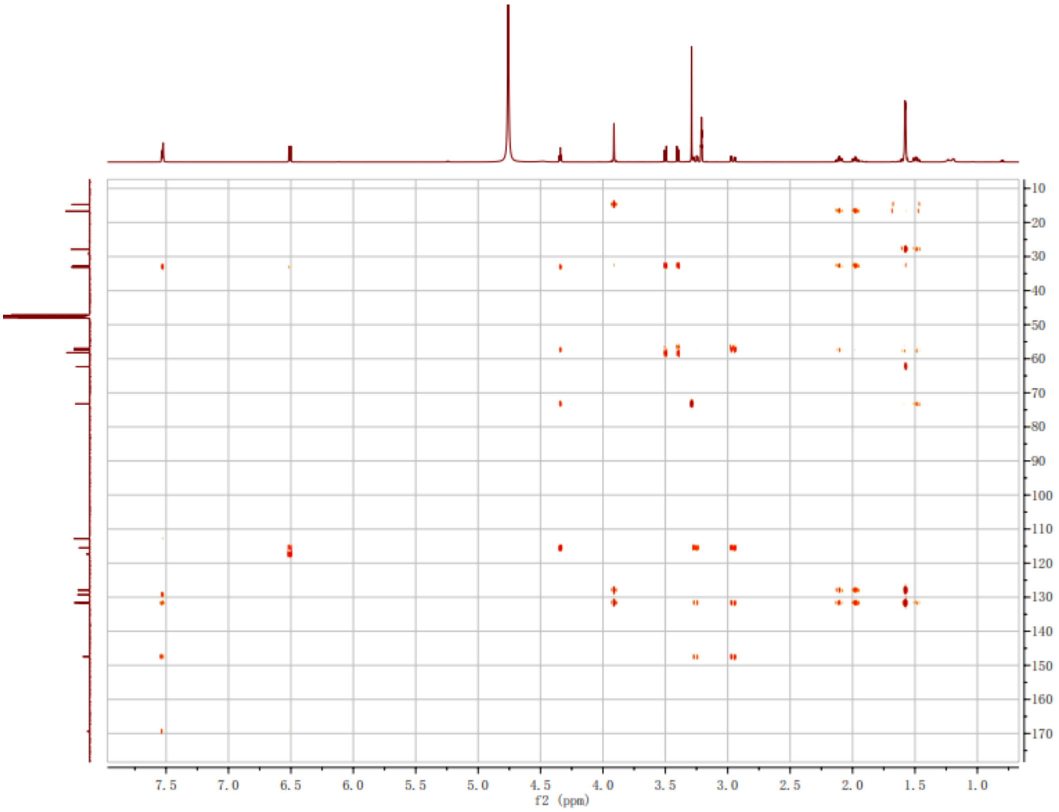

Figure S22

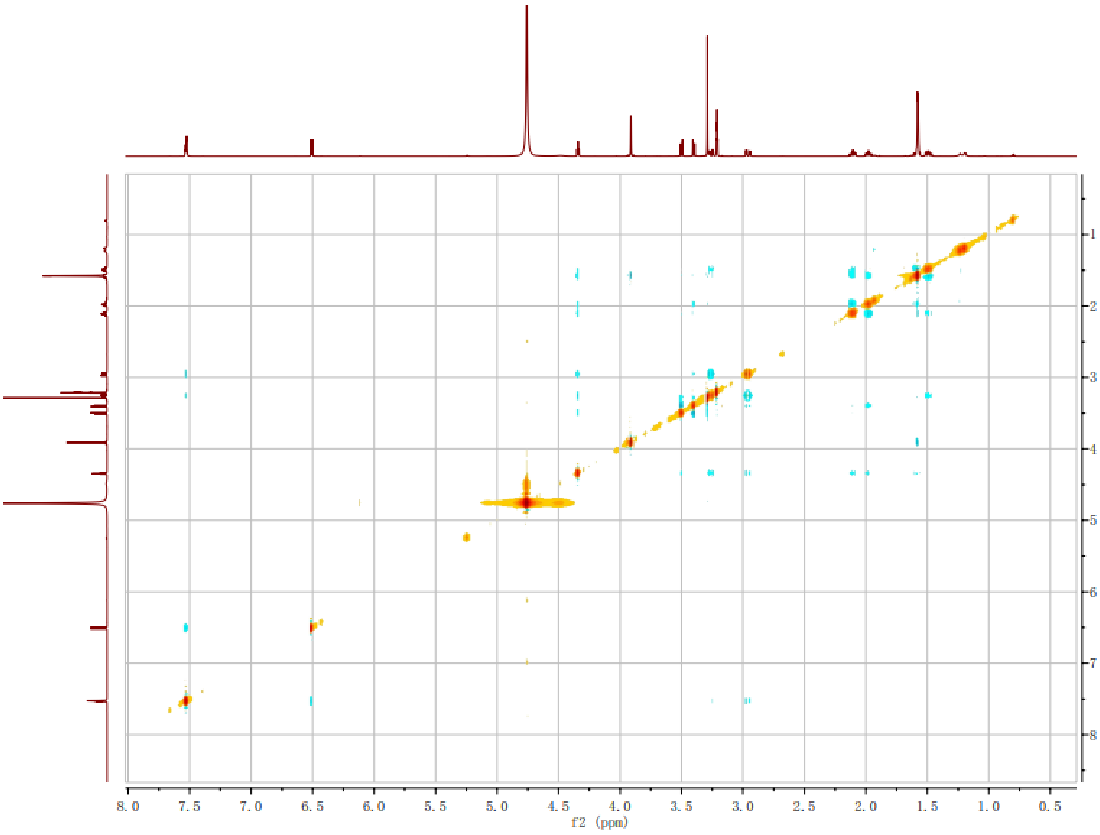

Figure S23

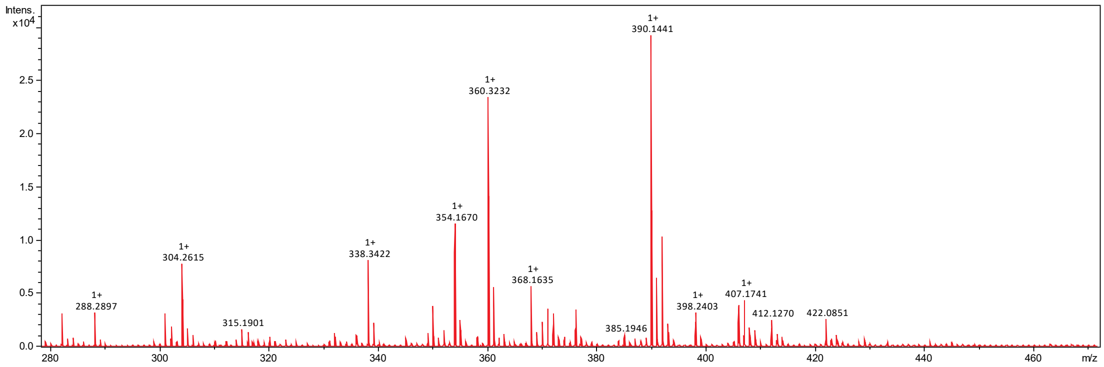

Figure S24

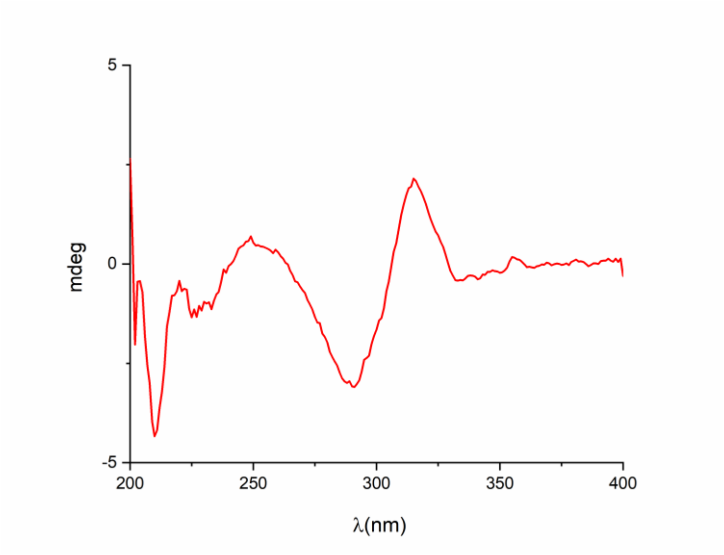

Figure S25

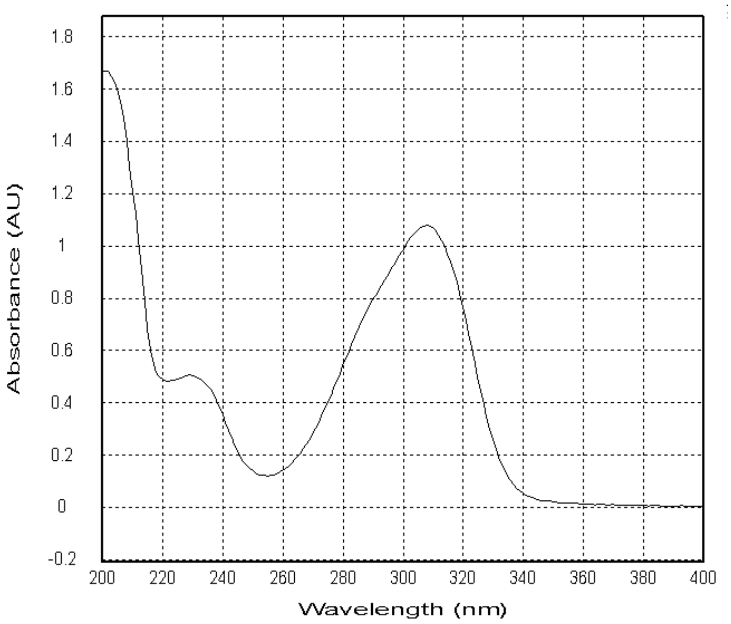

Figure S26

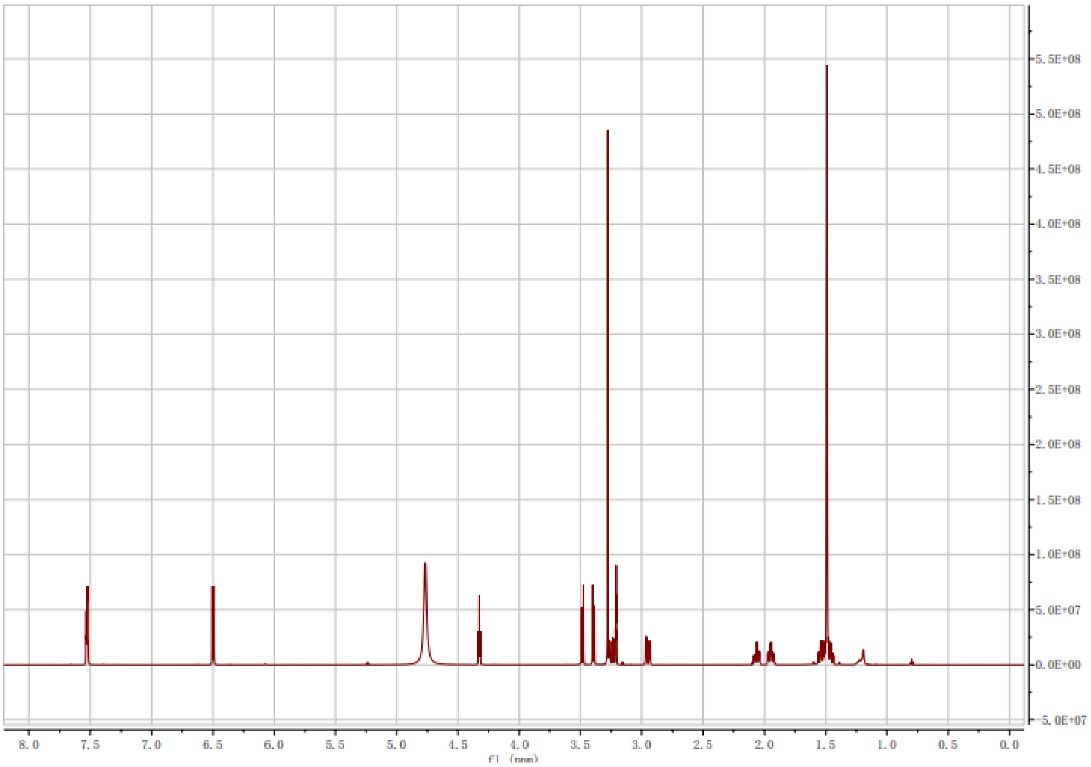

Figure S27

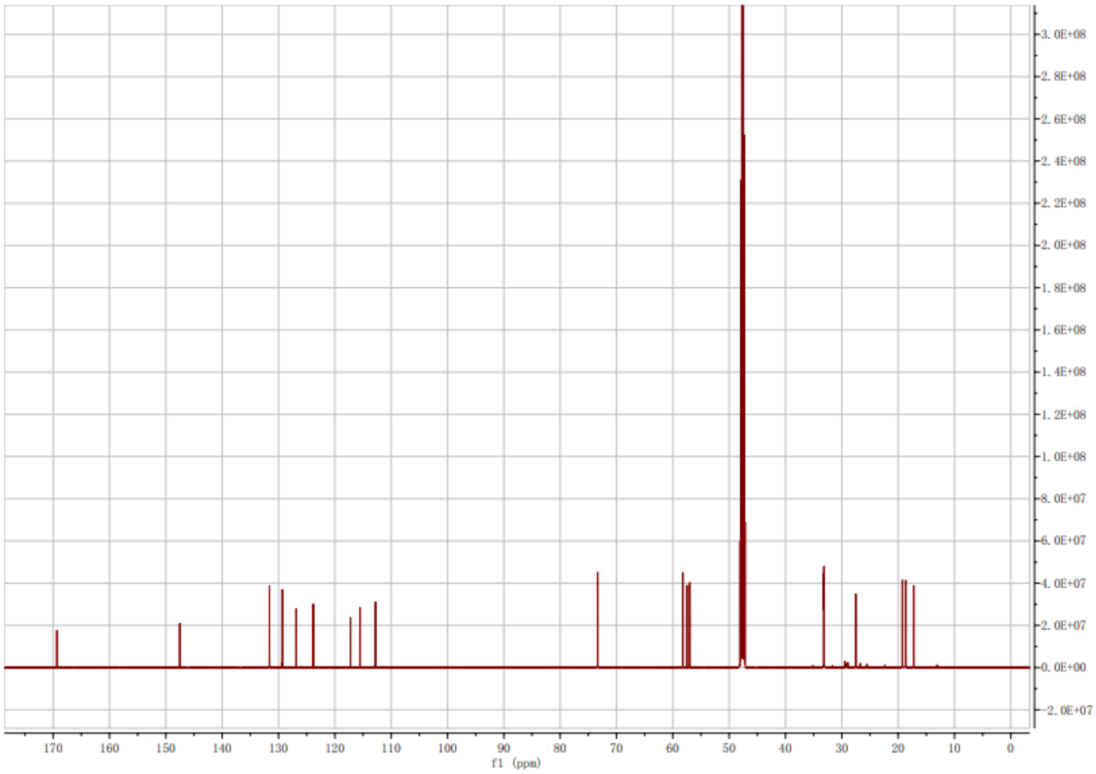

Figure S28

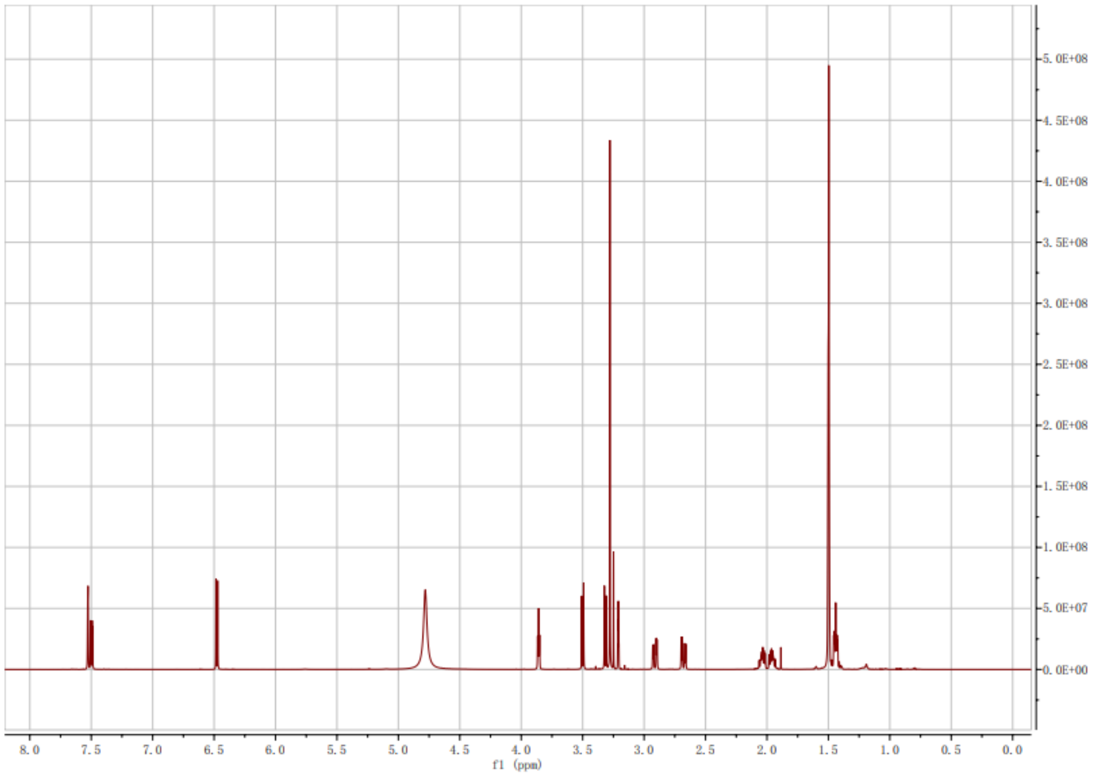

Figure S29

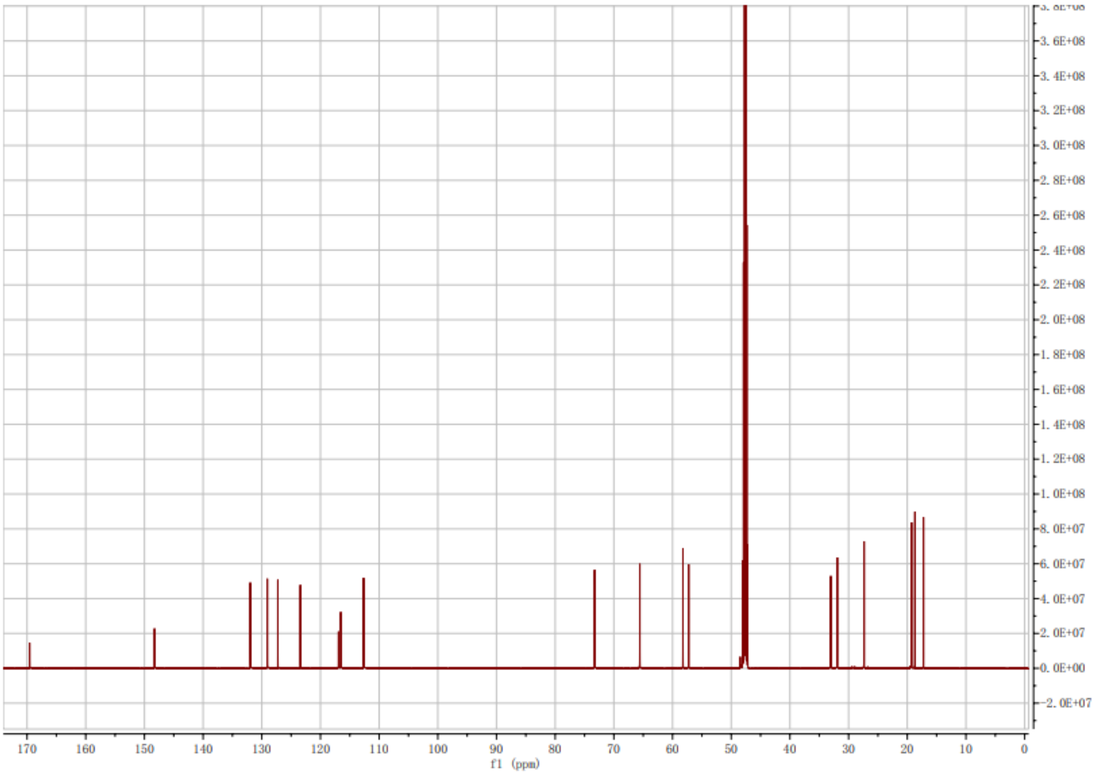

Figure S30

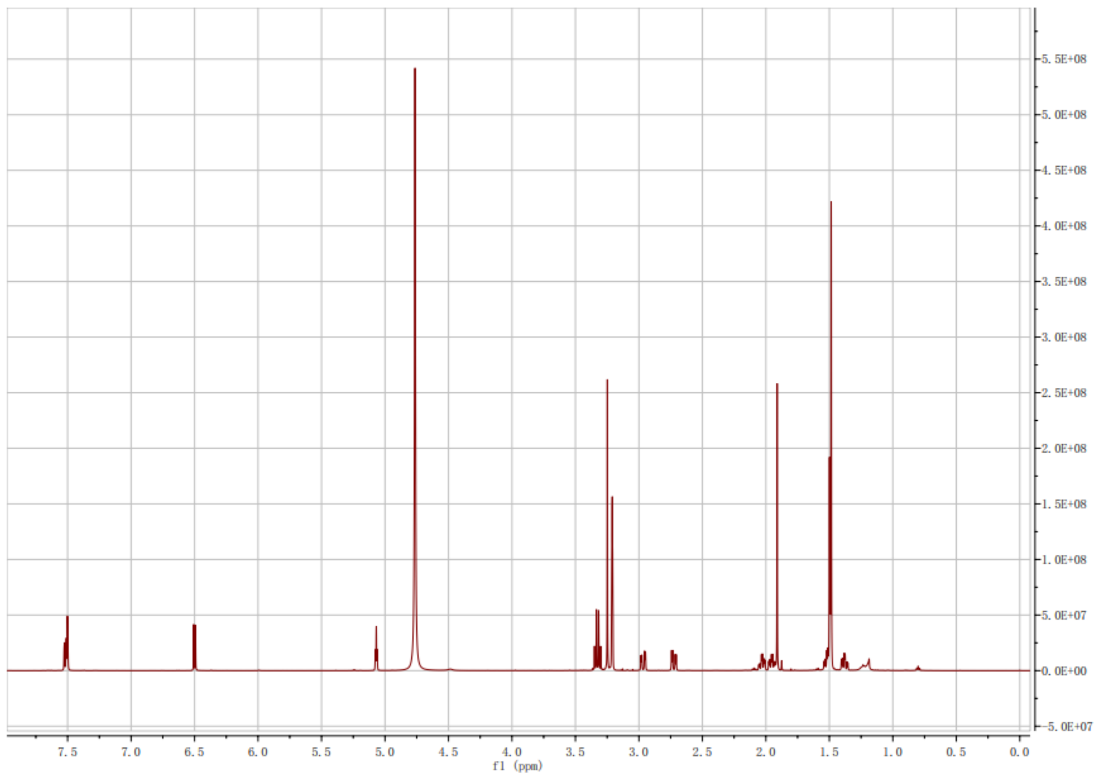

Figure S31

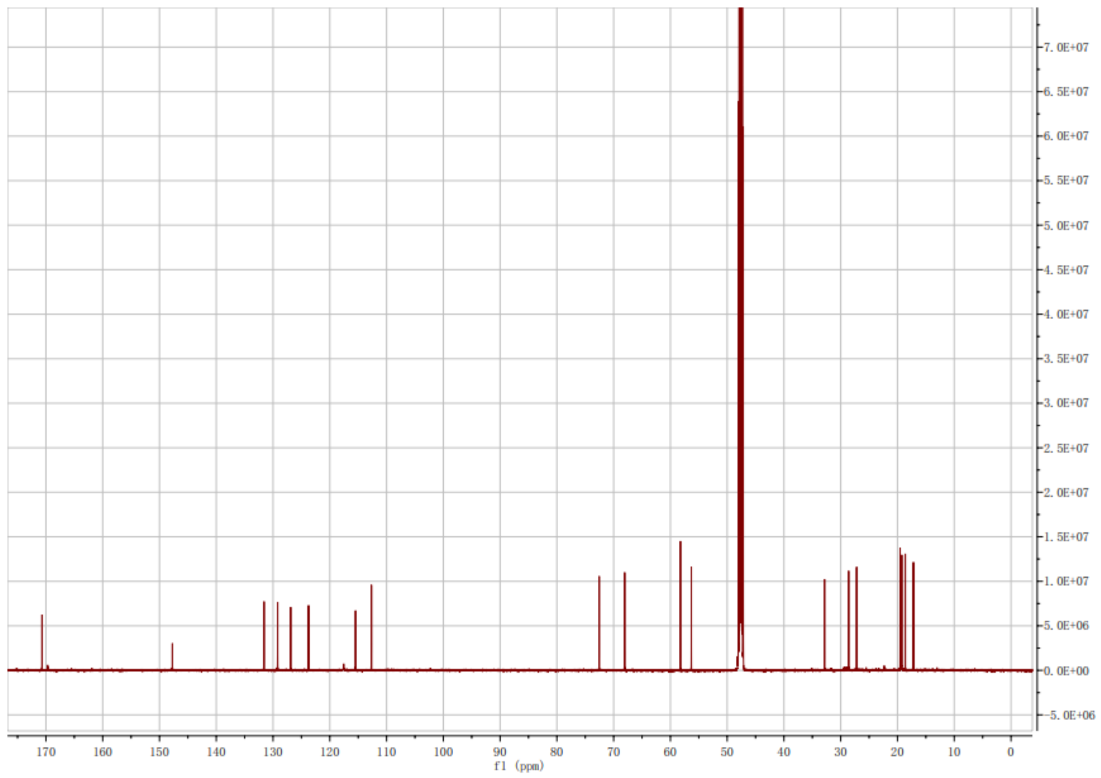

Figure S32

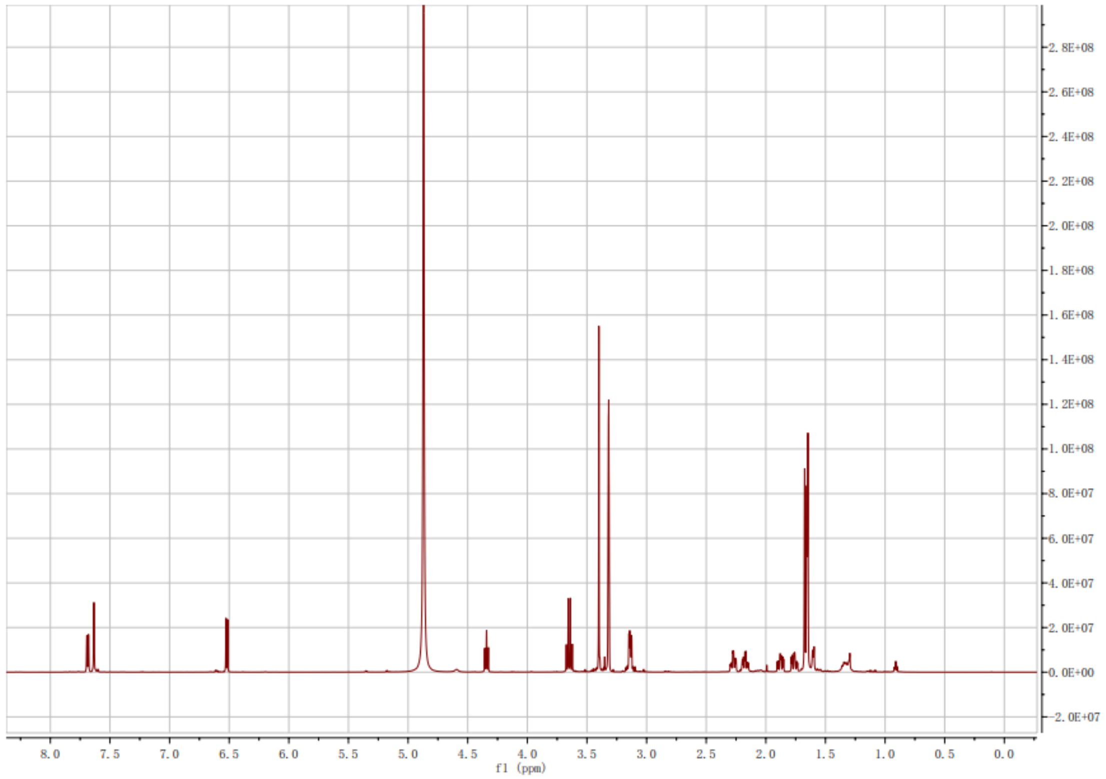

Figure S33

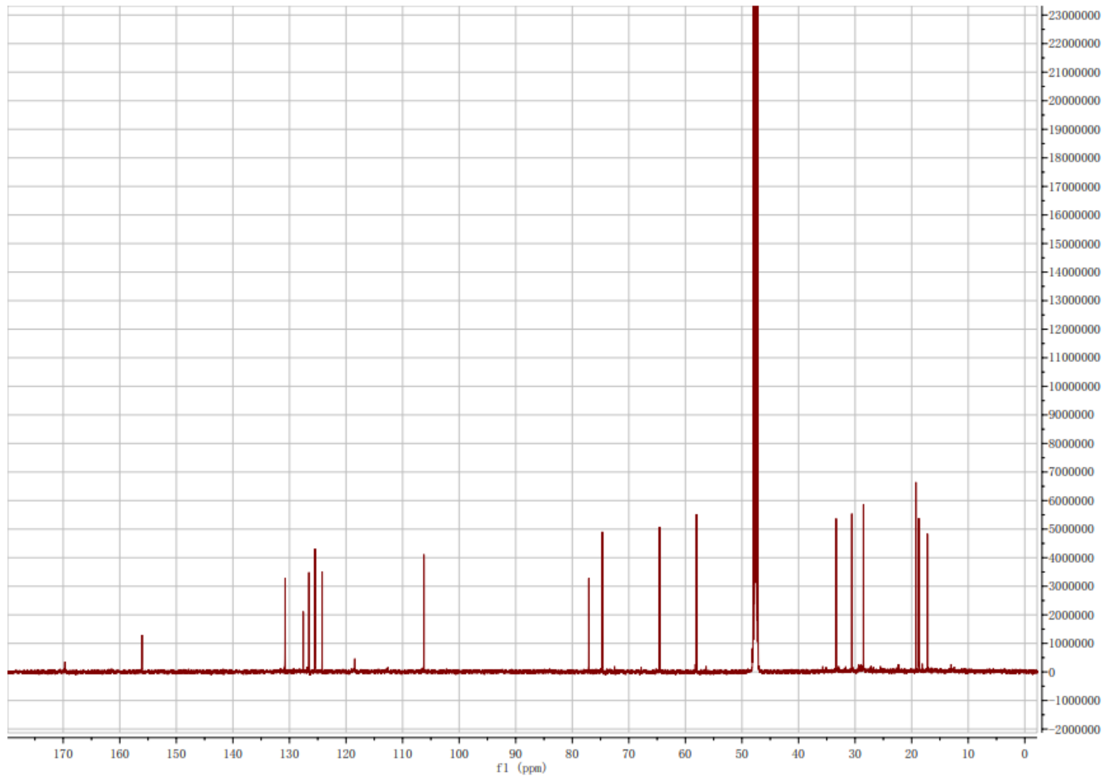

Figure S34

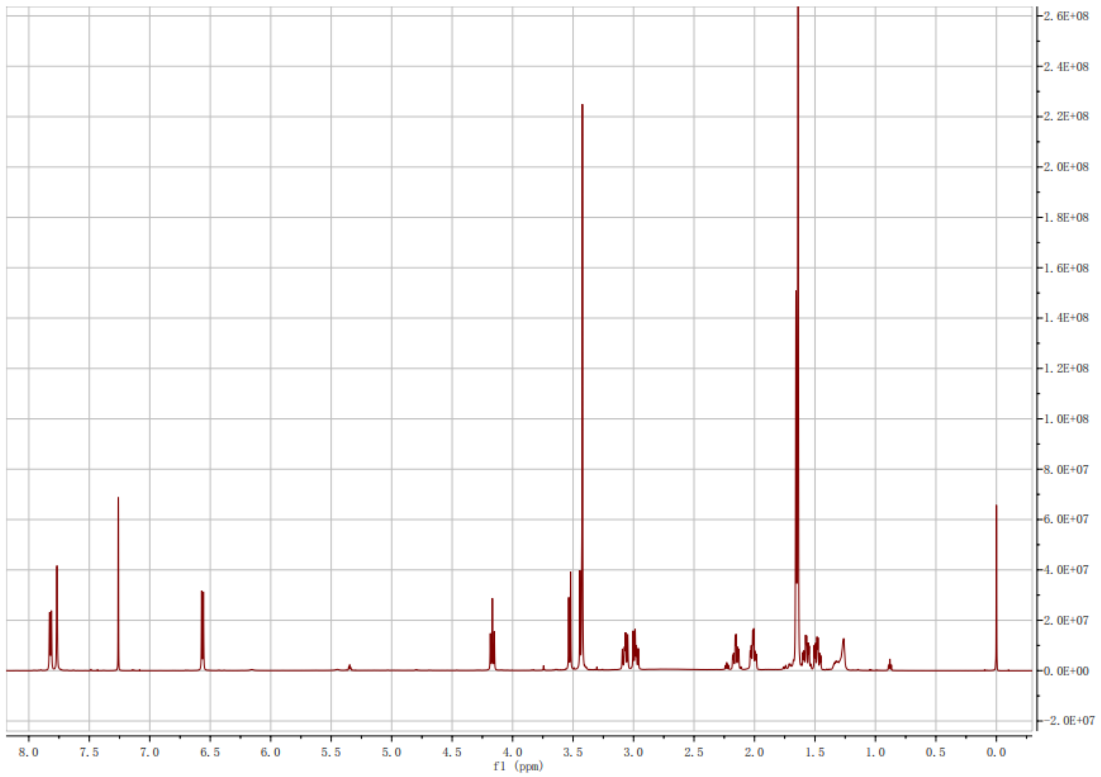

Figure S35

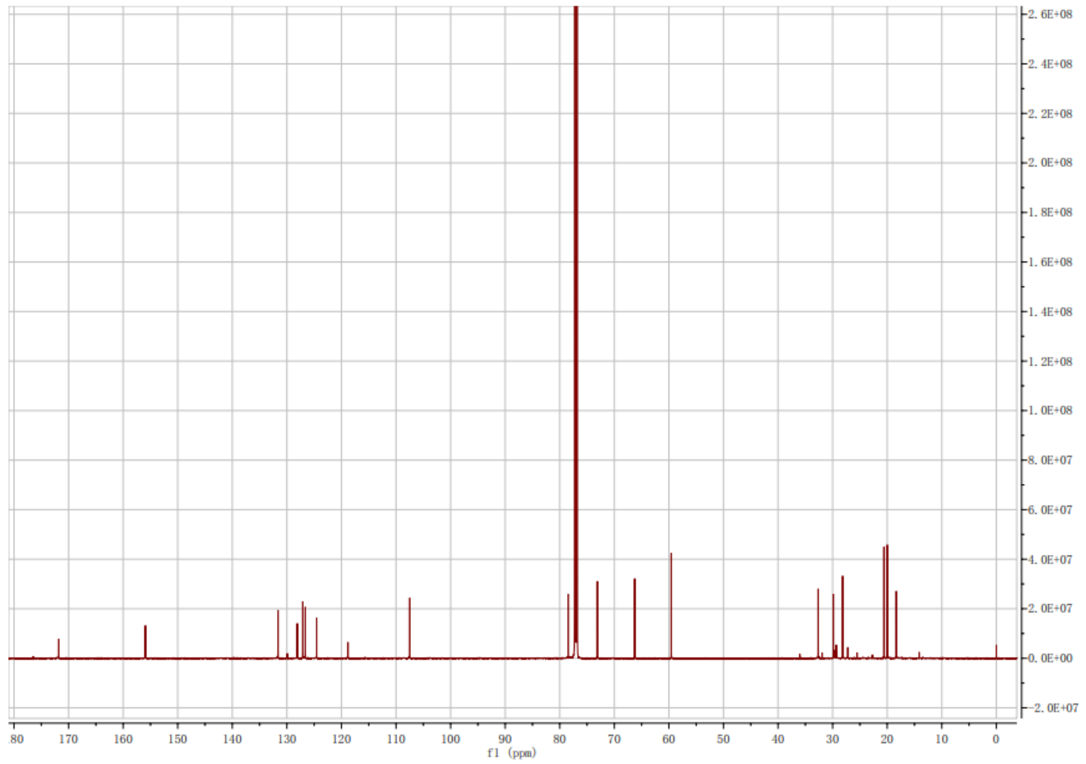

Figure S36
